# Digitally Based Blood Pressure Self-Monitoring Program That Promotes Hypertension Self-Management and Health Education Among Patients With Low-Income: Usability Study

**DOI:** 10.2196/46313

**Published:** 2023-07-24

**Authors:** Jacqueline Yareli Poblete, Natalie Lauren Vawter, Sydney Virginia Lewis, Earl Marc Felisme, Paloma Adriana Mohn, Jennifer Shea, Adam William Northrup, Jie Liu, Tala Al-Rousan, Job Gideon Godino

**Affiliations:** 1 Laura Rodriguez Research Institute Family Health Centers of San Diego San Diego, CA United States; 2 Herbert Wertheim School of Public Health and Human Longevity Science University of California San Diego San Diego, La Jolla, CA United States

**Keywords:** hypertension, blood pressure, digital health, health education, self-measured blood pressure monitoring, remote patient monitoring

## Abstract

**Background:**

According to evidence-based clinical guidelines, adults with hypertension are advised to self-monitor their blood pressure (BP) twice daily. Self-measured BP monitoring is a recommended strategy for improving hypertension management.

**Objective:**

We aimed to determine the feasibility and acceptability of a digitally based BP self-monitoring program that promotes hypertension self-management and health education among low-income patients. We hypothesized that the program would be highly feasible and acceptable and that at least 50% of the patients would use the monitor at the rate required for the reimbursement of the device’s cost (16 days of measurements in any 30-day period).

**Methods:**

Withings BPM Connect was deployed to patients at Family Health Centers of San Diego. Program elements included training, SMS text message reminders, and physician communication. Compliance, use, mean BP, and BP control status were calculated. A Kaplan-Meier time-to-event analysis was conducted to compare time to compliance between a strict definition (≥16 days in any rolling 30-day window) and a lenient definition (≥1 day per week for 4 consecutive weeks). A log-rank test was performed to determine whether the difference in time to compliance between the definitions was statistically significant. Mean systolic BP (SBP) and diastolic BP (DBP) before the intervention and after the intervention and mean change in SBP and DBP across patients were calculated. Paired sample *t* tests (2-tailed) were performed to assess the changes in SBP and DBP from before to after the intervention.

**Results:**

A total of 179 patients received the monitors. The mean changes in SBP and DBP from before to after the intervention were +2.62 (SE 1.26) mm Hg and +3.31 (SE 0.71) mm Hg, respectively. There was a statistically significant increase in both SBP and DBP after the intervention compared with before the intervention (*P*=.04 and *P*<.001). At the first and last measurements, 37.5% (63/168) and 48.8% (82/168) of the patients had controlled BP, respectively. During the observation period, 83.3% (140/168) of the patients had at least 1 controlled BP measurement. Use decreased over time, with 53.6% (90/168) of the patients using their monitor at week 2 and only 25% (42/168) at week 11. Although only 25.6% (43/168) achieved the strict definition of compliance, 42.3% (71/168) achieved the lenient definition of compliance. The median time to compliance was 130 days for the strict definition and 95 days for the lenient definition. The log-rank test showed a statistically significant difference in time to compliance between the compliance definitions (*P*<.001). Only 26.8% (45/168) complied with the measurement rate that would result in device cost reimbursement.

**Conclusions:**

Few patients used the monitors at a rate that would result in reimbursement, raising financial feasibility concerns. Plans for sustaining costs among low-income patients need to be further evaluated.

## Introduction

### Background

High blood pressure (BP), or hypertension, is one of the most prevalent health issues in the United States, affecting almost half of the adults (47% or 116 million) [1]. In the United States, 1 in 5 adults is unaware that they have hypertension, likely owing to a lack of symptoms [2], and only one-quarter of US adults with hypertension have the condition under control (<140/90 mm Hg) [1]. Untreated and uncontrolled hypertension raises the risk of heart disease, stroke, kidney damage, and other complications [3]. In 2020, hypertension was a main or contributing factor in >670,000 deaths in the United States [4]. Along with its impacts on morbidity and mortality, hypertension has a substantial economic burden, costing the United States between US $131 and US $198 billion per year (including the cost of health care services and BP medications) [5]. Patients with hypertension also have increased costs (>US $2000 more per year) compared with those without hypertension, including nearly double the annual prescription medication costs and 2.5 times the annual hospital expenses [5].

According to the Health Resources and Services Administration, 30 million Americans (1 in every 3 living in poverty and 1 in every 7 of a racial or ethnic minority group) receive medical care from federally qualified health centers (FQHCs) [6]. Although FQHCs provide preventive chronic disease and primary care services to medically underserved populations, uninsured individuals who visit an FQHC can still be charged for their care. The lack of health insurance is one of the most significant barriers to effective hypertension management among US adults [7]. Therefore, low-income and uninsured populations are the most susceptible to inadequate hypertension treatment and management. The current limited information on hypertension management in FQHCs highlights a need for further research in this patient population.

Hypertension management, including medication and lifestyle changes, can significantly reduce morbidity and mortality rates. Self-measured BP (SMBP) monitoring with clinical support is an evidence-based strategy shown to improve medication adherence and BP management [8]. In SMBP, a patient uses a BP monitor (BPM) to measure and record their BP readings outside of a clinical environment, usually at home [9]. This strategy has the potential to enhance the quality and accessibility of care for patients with hypertension. The use of SMBP that capitalizes on digitally connected devices allows measurements to be transferred to patients’ electronic health records (EHRs).

Despite SMBP being recommended as a successful strategy for hypertension management, there is a lack of infrastructure to enable proper SMBP transmission of BP measurements [10]. Patients encounter difficulties in sharing SMBP readings with providers outside of typical in-person visits, whereas providers face difficulties in incorporating SMBP data into their clinical workflow to provide timely patient feedback [10]. The digital management of hypertension through remote patient monitoring (RPM) supports SMBP transmission of BP readings. RPM is a technology that allows patients to be monitored outside of traditional clinical settings, such as at home, thereby increasing access to care and lowering health care delivery costs. Digital health and RPM can offer providers a more holistic perspective on their patients’ health and allow patients to take more control over their health.

### This Study

Few existing programs integrate SMBP with RPM to interpret BP data, encourage lifestyle changes, and titrate medication [11]. The purpose of this pilot study was to determine the feasibility and acceptability of a digitally based BP self-monitoring program that promotes hypertension self-management and health education among low-income patients. We hypothesized that the program would be highly feasible and acceptable to patients and that at least 50% of the patients would use their BPM at the rate required for the reimbursement of the device’s cost (16 days of measurements in any 30-day period).

## Methods

### Study Design and Study Population

This study was conducted at Family Health Centers of San Diego (FHCSD), the largest FQHC system in San Diego, California. FHCSD’s primary care clinics are strategically located in federally designated health professional shortage areas and serve medically underserved areas with high proportions of uninsured patients. FHCSD serves >160,000 unduplicated patients annually, the vast majority of whom are low-income individuals and members of a minority population. Approximately 32% are uninsured, 37% are best served in a language other than English, and >55% are Hispanic. The inclusion criteria for the digital health program were as follows: being an FHCSD patient who (1) was aged ≥18 years; (2) spoke English, Spanish, or Arabic; (3) had an appointment with FHCSD within the last 6 months; and (4) had a diagnosis or history of hypertension in their EHR. Exclusion criteria included not having completed an FHCSD Broad Consent form.

We deployed 180 cellularly connected BPMs to patients. The rolling enrollment period began in January 2022 and ended in July 2022. The monitors were deployed in two ways: (1) in person and (2) via the web. The goal was to deploy 90 devices remotely and 90 in person. By July, a total of 179 patients received a Withings BPM Connect, 90 (50.3%) of whom were remotely onboarded through Zoom (Zoom Video Communications, Inc) and 89 (49.7%) of whom were onboarded in person at clinics (1 monitor was lost during shipping).

### Training and Onboarding

During onboarding, each patient was trained on how to use the BPM and how to interpret BP readings. The patients also received health education on the importance of monitoring and managing hypertension.

The health education content that was provided to the participants included (1) background on SMBP, (2) how to properly prepare for self-measurement (eg, avoiding caffeine, smoking, and exercise from 30 minutes before measuring; waiting at least 30 minutes after a meal; and emptying bladder), (3) proper positioning for self-measurement (eg, feet flat on the floor, legs uncrossed, back straight, and arm supported or palm up), (4) how to use the BPM, (5) how to properly self-measure (eg, resting for 5 minutes before starting; relaxing the body; avoiding conversations, TV, or phones; and taking 2 to 3 measurements 1 minute apart), and (6) how to read and understand SMBP measurements and categories (normal, elevated, stage 1, stage 2, and hypertension crisis). We used the teach-back method to ensure that the patients understood the information provided and to address any additional questions or concerns from the patient.

### Measurement

Patients were advised to measure their BP at home twice per day, once in the morning and once in the late afternoon, per the American Heart Association’s (AHA) evidence-based guidelines for home BP monitoring. However, if they were unable to do so, we recommended that patients measure their BP at least every other day for 3 months using the Withings BPM Connect. This device is very user-friendly, is smaller than most BPMs, provides accurate measurements, is cellular enabled (ie, it can be used with or without a smartphone), and displays numerical readings with large LED lights and color-coded indicators [12].

Our recommended measurement plan of measuring at least every other day was based on the Centers for Medicare and Medicaid Services reimbursement requirement of at least 16 days of monitoring over a 30-day period [13]. Specifically, monitoring must occur on at least 16 days over a 30-day period for Current Procedural Terminology (CPT) codes 99453 and 99454 to be billed. CPT code 99453 is valued to reflect clinical staff time that includes instructing a patient about using one or more medical devices, and CPT code 99454 is valued to include the medical device supplied to the patient and programming of the medical device for repeated monitoring. Although measuring every other day during 30-day period equals 15/30 readings, assuming that the patients attempted to measure every day on occasion, this would have got us close to receiving at least 16/30 days of data within a 30-day period.

### Surveys

Patients were asked to complete a baseline survey to characterize digital health literacy and hypertension health outcomes, as well as a postprogram survey after 3 months of SMBP to gather feedback on the program and device usability (Multimedia Appendices 1 and 2). Baseline hypertension questions were developed with reference to the AHA and American Medical Association “Lower Your Blood Pressure” questionnaire, which collects information about medications, lifestyle changes, and challenges with managing BP [14]. Baseline digital health questions were developed using the Digital Health Literacy Instrument, which measures familiarity and the ability to operate digital devices [15].

For the postprogram survey, usability questions on the Withings BPM Connect were developed using the System Usability Scale (SUS), a 10-item survey with 5 response options ranging from strongly agree to strongly disagree [16]. This scale may be used to test a wide range of products and services, including hardware, software, mobile devices, websites, and apps [16]. The SUS items were modified to use a simpler language that our patient population could understand, and 2 of the items were removed. Item 6 (“I thought there was too much inconsistency in this system”) and item 8 (“I found the system very cumbersome to use”) were eliminated because of being redundant with other questions and having different meanings when translated into Spanish. In the 10-item SUS survey, scores >68 are considered above average [16]. In this study, the scoring multiplier was adjusted from 100/40 to 100/32 to accommodate the reduced number of questions [17].

### Clinical Support

To support healthy lifestyles and behavior change, educational booklets were created with colorful infographics clearly illustrating important BP concepts (Multimedia Appendix 3). The booklets included step-by-step instructions for taking accurate BP readings, information on readings and BP categories, and tips for success (eg, eat smart, move more, manage weight, do not smoke, and sleep well). The booklets were created in 3 languages: English, Spanish, and Arabic. Patients who were onboarded in person received a physical copy of the booklet, whereas patients who were onboarded remotely received a PDF version of the booklet through email.

To engage patients at home, an SMS text messaging campaign was offered (Multimedia Appendix 4). For 7 weeks, the participating patients received 1 SMS text message delivered at 9 AM daily. The SMS text messages were designed to remind patients to check their BP and to promote a healthier diet, physical activity, more sleep, stress awareness, and weight loss. All SMS text message content was evidence based and had previously been shown to stimulate behavior change [18,19].

After 3 months, when the postprogram survey was completed, the patients were asked whether they wished to continue using their BPM for another 3 months. We reassured them that we would continue to monitor their results and alert their providers of any elevated readings. If the patients agreed to continue using their monitor, they were sent monetary incentives (US $25 gift cards) via mail to thank them for continuing to use their devices and to encourage them to continue self-monitoring at home, with the goal of increasing measurement adherence even after program completion.

### Withings RPM

The Withings RPM was used to access and review patient data. This platform allows health care teams to create a customized measuring plan and collaborate with other professionals to manage multiple patients. The Withings RPM includes a feature that provides automated alerts for health care teams to review whenever a patient’s BP level is outside of the normal range. Measurement results (date, time, BP, and heart rate) were automatically sent to the platform, and FHCSD’s team of digital health specialists monitored the patients’ readings and alerts daily. If a patient continuously had elevated BP readings, their primary care provider was contacted via email with the suggestion to contact the patient and schedule a follow-up BP check visit. The protocol consisted of the following:

- If a patient triggered a yellow alert (≥160 mm Hg systolic BP [SBP] or ≥100 mm Hg diastolic BP [DBP]), the team monitored their measurements for the next 7 to 10 days. If the patient consistently had yellow alerts for those 7 to 10 days, the team then proceeded to send a message to the patient’s provider within 24 to 48 hours.
- If a patient triggered a red alert (≥180 mm Hg SBP or ≥120 mm Hg DBP), the team sent a message to their provider immediately within 24 to 48 hours. The team then continued to monitor their measurements for the next 7 to 10 days. If the patient consistently had red alerts for those 7 to 10 days, the team then proceeded to send an additional message to the patient’s provider within 24 to 48 hours.

The following are examples of the emails that were sent to providers.

This email was sent to providers via EHRs for consistent yellow alerts:

Hello, your patient has agreed to receive a digitally connected blood pressure monitor. We are monitoring patients’ readings and notifying their provider if readings are elevated. Your patient has been consistently having elevated blood pressure readings within the past week (>160mmHg SBP or >100mmHg DBP). We think that you or your team could contact them to schedule a follow-up blood pressure check appointment if you wish. Please feel free to reach out to us at digitalhealth@fhcsd.org if you have any questions. Thank you.

This email was sent to providers via EHRs for the first red alert:

Hello, your patient has agreed to receive a digitally connected blood pressure monitor. We are monitoring patients’ readings and notifying their provider if readings are too high. Your patient currently has out of range blood pressure readings (>180mmHg SBP or >120mmHg DBP). We think that you or your team could contact them to schedule a follow-up blood pressure check appointment if you so wish. Please feel free to reach out to us at digitalhealth@fhcsd.org if you have any questions. Thank you.

This email was sent to providers via EHRs for consistent red alerts:

Hello, this is a follow-up from my last email. Your patient has been consistently continuing to have out of range blood pressure readings within the past week (>180mmHg SBP or >120mmHg DBP). I am just sharing this again in case you or your team would like to schedule a follow-up blood pressure check appointment with them. Please feel free to reach out to us at digitalhealth@fhcsd.org if you have any questions. Thank you.

### Statistical Analysis

All data processing, analyses, and calculations were performed in R (version 4.1.2 Bird Hippie; R Foundation for Statistical Computing). Raw data from the BPMs were processed and cleaned, and patients who never activated their monitors were removed from the data set. Descriptive statistics were used to describe the demographic characteristics of the study population. Responses to the baseline digital health literacy survey questions were analyzed using descriptive statistics. Calculations related to compliance, use, mean BP, and BP control status were performed. Descriptive statistics were used to analyze the responses to the postprogram survey.

For the time-to-event analysis, a Kaplan-Meier analysis was conducted to compare time to compliance between 2 definitions of compliance: a strict definition that defined compliance as measuring BP on at least 16 days during any rolling 30-day window versus a lenient definition that defined compliance as measuring BP on at least 1 day per week for any 4 consecutive weeks [20]. For both definitions, measures on the day of onboarding were excluded. Each noncompliant patient was censored after their total number of days in the study. A log-rank test was performed to determine whether the difference in time to compliance between the definitions was statistically significant. An unadjusted (crude) Cox proportional hazards regression model was run to assess the time-to-event distributions by compliance definition group [21]. As the patient population was the same for both definitions, no additional variables (eg, demographic or clinical) could be added to the model; all of the effect would have been based on the definition. The proportional hazards assumption was tested using the *survival* package of R [22,23].

For the comparison of BP measurements taken before and after the intervention, BP data from FHCSD’s EHR system were obtained for a 12-month period prior to when each participant received the intervention. Mean preintervention SBP and DBP were calculated for each patient (except for patients with only 1 BP reading). For each patient, the preintervention mean was subtracted from the postintervention mean to calculate the changes in SBP and DBP. The mean changes in SBP and DBP across patients were calculated. The mean SBP and DBP before the intervention and after the intervention were also calculated across patients. Paired sample *t* tests (2-tailed) were performed to assess the changes in SBP and DBP from before to after the intervention. Statistical significance was defined as *P*<.05 for all analyses.

### Qualitative Survey Analysis

The participants were individually surveyed at the end of the program about their experience with the program and asked to numerically rate various aspects of the devices. At the end of this survey, the participants were asked open-ended questions to collect otherwise undiscussed feedback. Upon the completion of the study, these responses were reviewed, and broad, mutually exclusive themes were identified.

### Ethics Approval

This no more than minimal risk feasibility and acceptability study was conducted as a component of quality improvement activities at FHCSD, a covered entity [24], and there is no requirement for such activities to undergo review by an Institutional Review Board [25]. However, all patients aged >18 years must complete and sign FHCSD’s Broad Consent form to receive treatment. The Broad Consent form includes a specific authorization for the use of deidentified health information for population health and quality improvement studies. We have only included patients who have an up-to-date Broad Consent form within their EHR.

## Results

### Baseline Characteristics

Between January 2022 and July 2022, a total of 179 patients received BPMs at FHCSD. Of the 179 patients, 89 (49.7%) received a BPM and health education from a digital health specialist in person, and 90 (50.3%) received a BPM by mail and remote health education via Zoom. The demographic characteristics of the study population are shown in Table 1. Overall, the patients had a mean age of 55.1 (SD 12.0) years, and 57.5% (103/179) were women. Most patients were Hispanic (132/179, 73.7%) and reported Spanish as their preferred language (112/179, 62.6%). The most prevalent educational level was less than high school (74/179, 41.3%), followed by high school (63/179, 35.2%), and most participants (103/179, 57.5%) were unemployed.

All 179 patients were offered the SMS text messaging campaign; 120 (67%) opted into this campaign, and 59 (33%) declined. Of the 120 patients who opted into the SMS text message campaign, 45 (37.5%) were English speakers, and 75 (62.5%) were Spanish speakers.

At baseline, all 179 patients completed the high BP and digital health literacy survey. Table 2 shows the study population’s baseline digital health literacy question responses. Most patients reported owning a smartphone (141/179, 78.8%) and had access to the internet or Wi-Fi at home (132/179, 73.7%), whereas 59.2% (106/179) of the participants reported that they did not have a computer, a laptop, or an iPad (Apple, Inc) at home.

**Table 1:** Baseline characteristics (N=179).

Characteristic			Values
**Gender, n (%)**			
	Women	103 (57.5)	
	Men	76 (42.5)	
**Age (years)**			
	Values, mean (SD)	55.1 (12.0)	
	Values, median (range)	56.0 (26.0-84.0)	
**Age group (years), n (%)**			
	18-29	4 (2.2)	
	30-39	15 (8.4)	
	40-49	33 (18.4)	
	50-59	58 (32.4)	
	≥60	69 (38.5)	
**Race, n (%)**			
	Asian	4 (2.2)	
	Black	20 (11.2)	
	White	151 (84.4)	
	Multiracial	4 (2.2)	
**Ethnicity, n (%)**			
	Hispanic	132 (73.7)	
	Non-Hispanic	46 (25.7)	
	Unknown	1 (0.6)	
**Education level, n (%)**			
	Less than high school	74 (41.3)	
	High school	63 (35.2)	
	College graduate	18 (10.1)	
	Postgraduate	4 (2.2)	
	Unknown	20 (11.2)	
**Employment status, n (%)**			
	Employed	63 (35.2)	
	Unemployed	103 (57.5)	
	Retired	13 (7.3)	
**Preferred language, n (%)**			
	English	61 (34.1)	
	Spanish	112 (62.6)	
	Other	6 (3.4)	
**Deployment type, n (%)**			
	In person	89 (49.7)	
	Remote	90 (50.3)	

**Table 2:** Baseline digital health literacy question responses (N=179).

Question and response			Participants, n (%)
**Do you have a smartphone?**			
	Yes	141 (78.8)	
	No	38 (21.2)	
**Do you have a computer, laptop, or iPad at home?**			
	Yes	73 (40.8)	
	No	106 (59.2)	
**Do you have internet and/or Wi-Fi at home?**			
	Yes	132 (73.7)	
	No	46 (25.7)	
	Missing	1 (0.6)	

### Compliance

Of the 179 patients who received a BPM, 168 (93.9%) activated their devices, and 11 (6.1%) did not. Approximately 6 months after rolling enrollment began in January 2022, a total of 26.8% (45/168) of the patients were compliant with the recommended measuring plan of 16 out of 30 days, the measurement rate that would result in device cost reimbursement. Most patients (123/168, 73.2%) did not achieve this target.

### Use of BP Monitors

**Figure 1:**
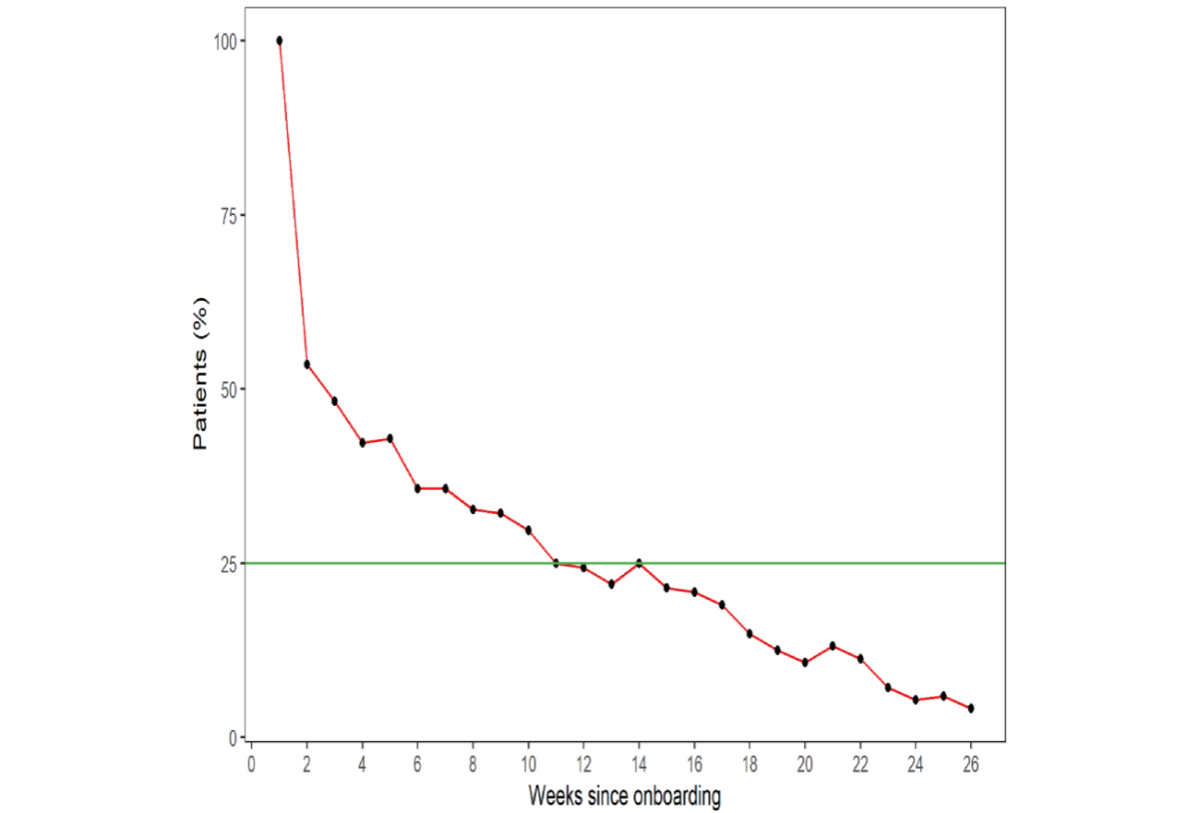
Use of the blood pressure monitors over time (n=168). Patients participated in the program for different durations, with some participating for <26 weeks.

Figure 1 illustrates the percentage of patients who used their BPMs for >26 weeks (approximately 6 months) after rolling enrollment began in January 2022. On the day of onboarding, the digital health specialist took 1 measurement for each patient, resulting in a 100% (n=168) use rate for week 1 (week 1 was defined as the first week of BP measurement for each patient). The use of the BPMs decreased over time, with 53.6% (90/168) of the patients using their monitor at week 2, a total of 42.3% (71/168) using their monitor at week 4, and 35.7% (60/168) using their monitor at week 6. At week 11, a total of 25% (42/168) of the patients were using their monitor; at week 26, the use rate was 4.2% (7/168).

### BP Control

**Figure 2:**
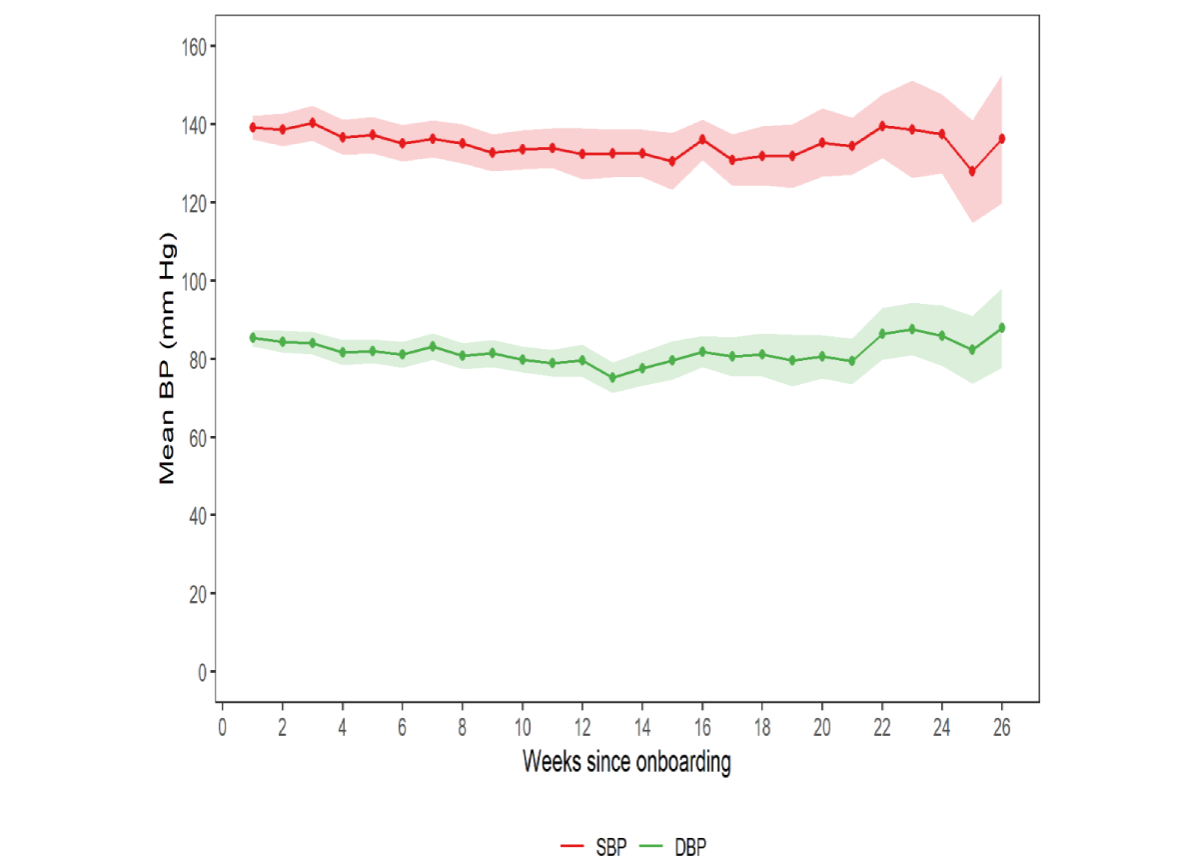
Mean systolic blood pressure (SBP) and diastolic blood pressure (DBP) by week (n=168). Shaded regions represent 95% CI for weekly means. BP: blood pressure.

Figure 2 illustrates the mean SBP and DBP across participants by week, with the number of participants decreasing over time (n=168 at week 1; n=7 at week 26). Both measures were consistent over time. Overall, the mean SBP was 136.2 (SD 19.6) mm Hg, and the mean DBP was 82.2 (SD 13.6) mm Hg. Over the entire study period, there were 913 yellow alerts (15.4% of all 5935 readings) and 75 red alerts (1.3% of all 5935 readings).

**Figure 3:**
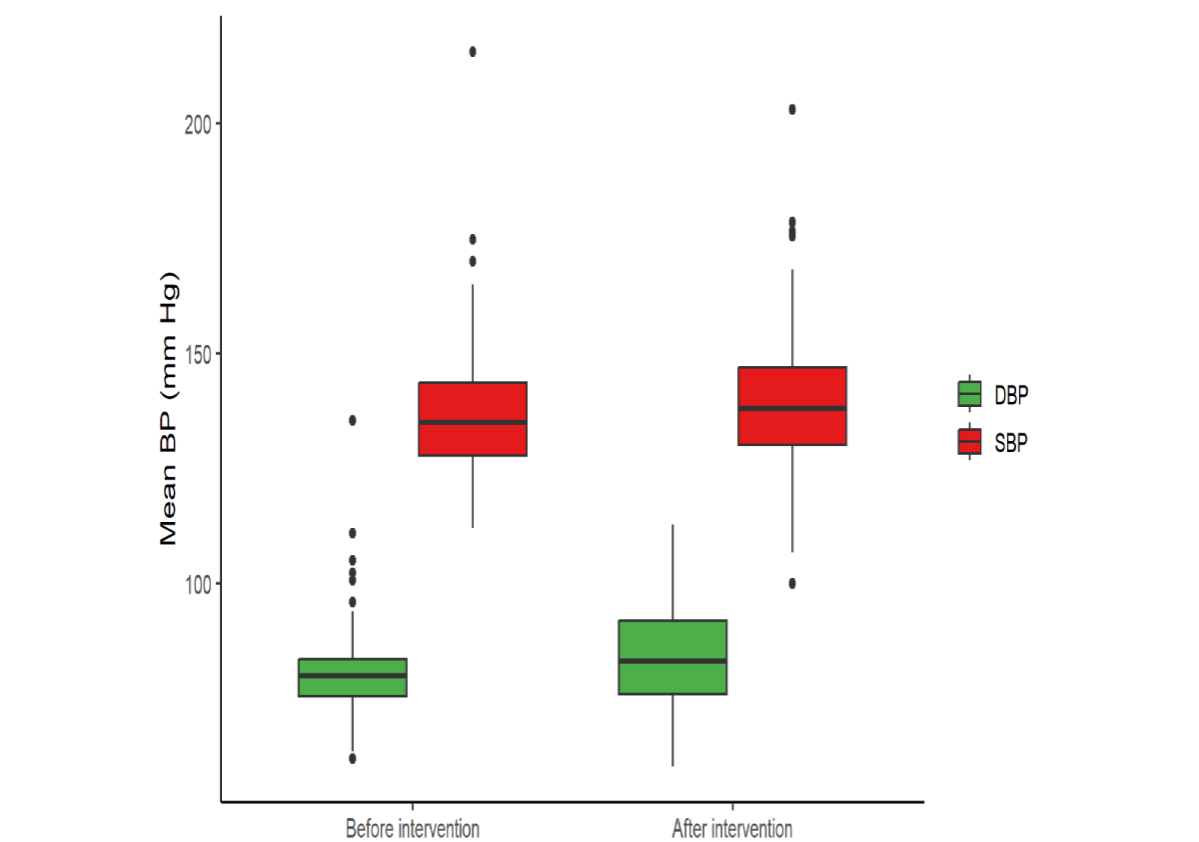
Mean systolic blood pressure (SBP) and diastolic blood pressure (DBP) before and after the intervention (n=154). BP: blood pressure.

Preintervention BP data from EHRs were available for 154 (86%) out of 179 patients. The mean change in mean SBP from before to after the intervention was +2.62 (SE 1.26) mm Hg. The mean change in mean DBP from before to after the intervention was +3.31 (SE 0.71) mm Hg. Overall, 42.2% (65/154) of the patients had a decrease in SBP following the intervention, and 57.8% (89/154) of the patients had an increase in SBP following the intervention, whereas 30.5% (47/154) of the patients had a decrease in DBP following the intervention, and 69.5% (107/154) of the patients had an increase in DBP following the intervention. The mean SBP and DBP before and after the intervention are shown in Figure 3. The paired sample *t* test (2-tailed) conducted to assess the change in SBP from before to after the intervention produced the following results (*t*_153_=2.0762; *P*=.04). The paired sample *t* test conducted to assess the change in DBP from before to after the intervention produced the following results (*t*_153_=4.6702; *P*<.001). For both *t* tests, the *P* value was <.05, indicating that there was a statistically significant increase in both SBP and DBP after the intervention compared to before the intervention.

Table 3 shows the participants’ BP control status at the first, last, and any measures—37.5% (63/168) of the patients were in control at their first BP measure, and 48.8% (82/168) were in control at their last measure. Although 83.3% (140/168) of the patients had at least 1 measure in control, 16.7% (28/168) did not achieve control at any measure.

**Table 3:** Blood pressure control status at first, last, and any measures (n=168).

	Uncontrolled, n (%)	Controlled, n (%)
First measure	105 (62.5)	63 (37.5)
Last measure	86 (51.2)	82 (48.8)
Any measure	28 (16.7)	140 (83.3)

### Time to Compliance

**Figure 4:**
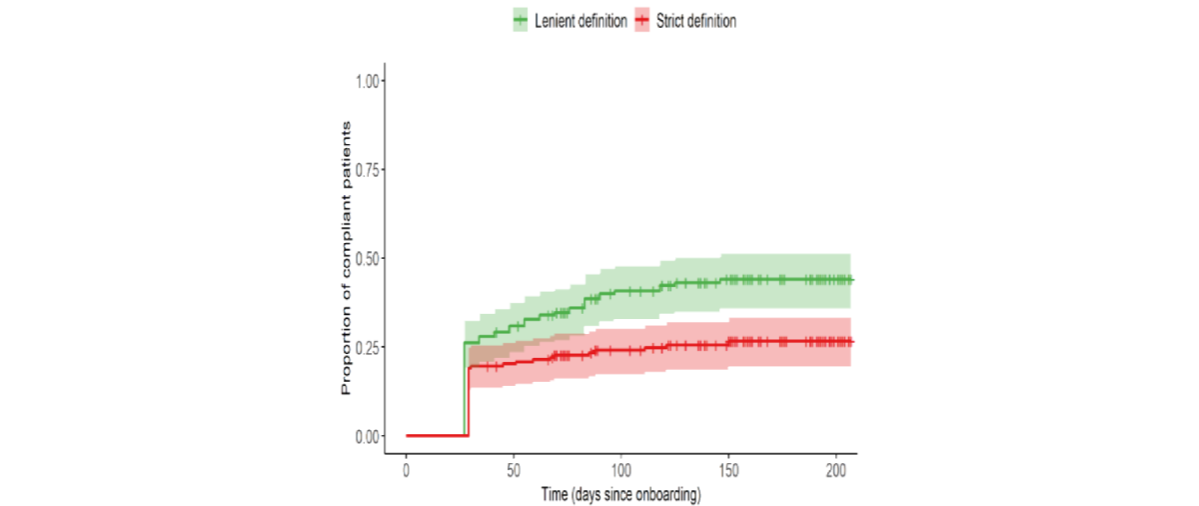
Time to compliance by the definition of compliance (n=168). Lenient definition: measured ≥1 day per week for 4 consecutive weeks. Strict definition: measured ≥16 days during any rolling 30-day window. The tick marks represent censored patients.

Time to compliance was compared between 2 definitions of compliance: a strict definition that defined compliance as measuring BP on at least 16 days during any rolling 30-day window and a lenient definition that defined compliance as measuring BP on at least 1 day per week for any 4 consecutive weeks. On the basis of the strict definition, 25.6% (43/168) of patients achieved compliance, and 74.4% (125/168) of patients did not achieve compliance. For the strict definition, the mean time to compliance was 119 days, and the median time to compliance was 130 (range 29-207) days. On day 29, the first opportunity, 32 (19%) of 168 patients met the strict definition of compliance. On the basis of the lenient definition, 42.3% (71/168) of patients achieved compliance, and 57.7% (97/168) of patients did not achieve compliance. For the lenient definition, the mean time to compliance was 103 days, and the median time to compliance was 95 (range 27-207) days. On day 27, the first opportunity, 26.2% (44/168) of patients met the lenient definition of compliance. Figure 4 shows the time to compliance for each definition. The log-rank test showed that the difference in time to compliance between the compliance definitions was statistically significant (*P*<.001). The unadjusted (crude) Cox proportional hazards regression model showed that there was a statistically significant difference in time to event between the compliance definitions (hazard ratio=0.51; *P*<.001, for the strict definition compared with the lenient definition; test for the proportional hazards assumption had a *P* value of .007).

### Feasibility and Acceptability

At follow-up, 83.2% (149/179) of patients completed the postprogram survey. Of the 179 patients who completed the baseline survey, 30 (16.8%) were lost to follow-up (≥4 call attempts with no answer or phone number disconnected). The postprogram survey responses, presented in Table 4, indicate a high level of feasibility and acceptability. When asked to rate the BPM, over three-quarters (113/149, 75.8%) of the participants selected “very good” (55/149, 36.9%) or “good” (58/149, 38.9%). Furthermore, 91.9% (137/149) of the participants reported that they would recommend the BPM to others. A very small percentage of participants (3/149, 2%) rated the BPM as either “bad” or “very bad,” and 71.8% (107/149) of the participants reported that they would be willing to continue using the BPM for another 3 months.

In the postprogram survey, the participants were asked 8 questions from the SUS. The mean score was 62.7 (SD 16.6), and the median score was 68.8 (Table 5). This corresponds to a qualitative interpretation of the patients scoring Withings BPM Connect as average in regard to effectiveness, efficiency, and satisfaction. The patients were also prompted to provide open-ended feedback using 2 questions: “Do you think the blood pressure monitor is helping you better manage your hypertension?” and “Please feel free to share with us any other feedback you might have.” In answering these questions, many patients provided context for their decreased or discontinued use of the BPM. Table 6 details the prevalence of the most common responses. The most frequent barriers to device use were related to inconvenience due to medical, professional, or personal obligations. Many patients also reported challenges in operating the device, concerns about accuracy, the lack of medical need, forgetfulness, lost or stolen devices, and fear as reasons for not regularly using their BPM.

**Table 4:** Postprogram survey responses (n=149).

Question and response			Participants, n (%)
**How would you rate the blood pressure monitor?**			
	Very good	55 (36.9)	
	Good	58 (38.9)	
	Neutral	33 (22.1)	
	Bad	2 (1.3)	
	Very bad	1 (0.7)	
**Would you recommend the blood pressure monitor to other patients?**			
	Yes	137 (91.9)	
	No	12 (8.1)	
**Are you willing to continue using the blood pressure monitor for another 3 months?**			
	Yes	107 (71.8)	
	No	42 (28.2)	

**Table 5:** System Usability Scale (SUS) analysis scores (n=149).

			Values
**Calculated SUS score**			
	Values, mean (SD)	62.7 (16.6)	
	Values, median (range)	68.8 (18.8-93.8)	
**I think that I would like to use the Withings BPM^a^ frequently, n (%)**			
	Strongly agree	32 (21.5)	
	Agree	47 (31.5)	
	Neutral	45 (30.2)	
	Disagree	23 (15.4)	
	Strongly disagree	1 (0.7)	
	Missing	1 (0.7)	
**I found the Withings BPM complicated to use, n (%)**			
	Strongly agree	8 (5.4)	
	Agree	16 (10.7)	
	Neutral	25 (16.8)	
	Disagree	73 (49)	
	Strongly disagree	27 (18.1)	
**I thought the Withings BPM was easy to use, n (%)**			
	Strongly agree	31 (20.8)	
	Agree	73 (49)	
	Neutral	33 (22.1)	
	Disagree	10 (6.7)	
	Strongly disagree	2 (1.3)	
**I think that I would need the support of a digital health specialist to use the Withings BPM, n (%)**			
	Strongly agree	23 (15.4)	
	Agree	22 (14.8)	
	Neutral	34 (22.8)	
	Disagree	67 (45)	
	Strongly disagree	3 (2)	
**I found the Withings BPM’s measurements easy to read, n (%)**			
	Strongly agree	51 (34.2)	
	Agree	74 (49.7)	
	Neutral	21 (14.1)	
	Disagree	3 (2)	
**I would imagine that most people would learn to use the Withings BPM very quickly, n (%)**			
	Strongly agree	3 (2)	
	Agree	89 (59.7)	
	Neutral	39 (26.2)	
	Disagree	18 (12.1)	
**I felt very confident using the Withings BPM, n (%)**			
	Strongly agree	11 (7.4)	
	Agree	77 (51.7)	
	Neutral	37 (24.8)	
	Disagree	20 (13.4)	
	Strongly disagree	4 (2.7)	
**I needed to learn a lot of things before I could use the Withings BPM, n (%)**			
	Strongly agree	12 (8.1)	
	Agree	47 (31.5)	
	Neutral	34 (22.8)	
	Disagree	52 (34.9)	
	Strongly disagree	4 (2.7)	

^a^BPM: blood pressure monitor.

**Table 6:** Feedback from the patients on the usability of the blood pressure monitor (n=149).

Reason for decreased or discontinued use	Prevalence, n (%)	Examples
Inconvenience in using the device	41 (27.5)	Prioritizing another health condition Busy schedule due to personal or professional obligations
Challenge in operating the device	36 (24.2)	Cannot use the device while alone, as help from family is required to use the device Physical discomfort in using the device, as it does not fit or causes Technical issue, namely device malfunction or confusion in operating the device
Concerns about accuracy	16 (10.7)	Results do not match the readings of other blood pressure monitoring devices. Results do not reflect the patient’s expectations.
Belief that use was not medically necessary	15 (10.1)	—^a^
Forgetting to use the device	14 (9.4)	—
Lost or stolen device or charger	10 (6.7)	—
Fear of viewing worrisome results	8 (5.4)	—

^a^Not available.

## Discussion

### Principal Findings

Results of the baseline digital health literacy survey revealed that most patients owned a smartphone (141/179, 78.8%) and had access to the internet or Wi-Fi at home (132/179, 73.7%), but less than half (73/179, 40.8%) of the participants had a computer, a laptop, or an iPad at home. Before implementing this program, we hypothesized that at least 50% of the patients would use their monitor at the rate required for the reimbursement of the cost of the device (16 days of measurements in any 30-day period). Approximately 6 months after the beginning of rolling enrollment in January, use and compliance data did not support this hypothesis. Only 27% (45/168) of the patients complied with the measurement plan that would result in device cost reimbursement, whereas 73% (123/168) of the patients did not achieve this target. The fact that such a low proportion of patients complied with the recommended measurement rate that would result in device cost reimbursement suggests that the current criteria for reimbursement may be too stringent and, therefore, inappropriate in lower resource settings. Furthermore, if a patient’s BP is under control, it is instinctual to reduce the frequency of home measures. Therefore, the likelihood of compliance with a reimbursable measurement threshold may decrease with increased BP control. It is critical for those creating the logic behind the financial support of RPM programs to consider this potential.

The use of BPMs declined over time, with use rates of 53.6% (90/168) at week two, 42.3% (71/168) at week four, 35.7% (60/168) at week six, 25% (42/168) at week 11, and 4.2% (7/168) at week 26. There was a slight but statistically significant increase in both SBP and DBP following the intervention. Only 25.6% (43/168) of the patients achieved the strict definition of compliance (measuring at least 16 days out of any 30 consecutive days), whereas 42.3% (71/168 of the patients achieved the lenient definition (measuring at least 1 day per week for 4 consecutive weeks). The difference in time to compliance between these compliance definitions was statistically significant (*P*<.001). With respect to BP control status, 37.5% (63/168) of the patients were in control at their first measurement, 48.8% (82/168) were in control at their last measurement, and 83.3% (140/168) had at least 1 measurement in control. In total, 16.7% (28/168) of the patients did not achieve control at any measurement. This observation has important implications for the definitions of clinical quality. Specifically, the Uniform Data System and Health Care Effectiveness Data and Information Set define hypertension control as SBP <140 mm Hg and DBP <90 mm Hg [26,27]. Furthermore, during a calendar year, every patient aged 18 to 85 years with a hypertension diagnosis should have a controlled BP reading documented during a qualified medical visit. When assessing the compliance with the Uniform Data System or Health Care Effectiveness Data and Information Set measure of hypertension control, only the last BP reading in a patient’s medical record in a calendar year is considered [26,27]. In the context of RPM, a new BP reading can appear in a patient’s medical record on a daily basis. This raises fundamental questions about the operational definition of quality when it comes to hypertension control. Our numbers indicated that the potential compliance differs by as much as 46%, depending on what is chosen as the last observation. With this in mind, researchers, scientists, clinicians, and policy makers must come together to further explore the implications of RPM and its impact on clinical quality.

We also hypothesized that our digital health program would have high feasibility and acceptability among our study population. At follow-up, the postprogram survey results corroborated this hypothesis, with most participants (113/149, 75.8%) rating the BPM as either “very good” or “good” and a very small percentage of participants (3/149, 2%) rating the BPM as either “bad” or “very bad.” Most participants reported that they would recommend the BPM to others (137/149, 91.9%) and that they would be willing to continue using the BPM for another 3 months (107/149, 71.8%). These results indicate a high level of acceptability, which is interesting given the low use and compliance rates observed over time. Although the patients recommended these devices to others and positively rated their experience, the additional feedback offered at the end of the survey revealed several barriers that made it challenging for them to use their BPM. The patients reported experiencing issues with convenience, technical challenges, and concerns about accuracy. Although patients seemed to recognize the benefits of the BPM, these issues may have outweighed their interest in continued regular use. This feedback provides an interesting context for the disparity between our positive survey results and the low use and compliance rates.

### Comparison With Prior Work

The effectiveness of RPM programs at improving the management of chronic diseases, including hypertension, has been established. A 2022 overview of recent systematic reviews used the Grading of Recommendations Assessment, Development, and Evaluation approach to evaluate the certainty of the evidence among randomized controlled trials using RPM in adult patients with hypertension, diabetes, or both [28]. The findings suggested that RPM likely caused a small decrease in SBP, although it was unclear whether this decrease was clinically meaningful [28]. The Community Preventive Services Task Force reported finding sufficient evidence of the effectiveness of SMBP interventions when used alone but strong evidence of the effectiveness of SMBP interventions when combined with additional support (eg, patient education, counseling, or web-based support) [29].

The effectiveness of RPM programs at improving hypertension management, specifically in underserved patient populations, has previously been explored, with promising findings. A study assessing the effectiveness of SMBP among medically underserved, low-income Black and Hispanic patients with hypertension reported a decrease in both SBP and DBP at follow-up [30]. A pilot study implementing the Measure Accurately, Act Rapidly, and Partner With Patients evidence-based protocol to address hypertension among a medically underserved patient population identified a clinically and statistically significant improvement in hypertension control 6 months after the intervention [31]. A study in Hispanic adults with uncontrolled hypertension analyzed the efficacy of a culturally tailored, smartphone-enabled self-management program that included SMBP and reported clinically and statistically significant reductions in SBP following the intervention [32]. A pilot study assessing the use of a tailored mobile health intervention in Black patients with hypertension and type 2 diabetes reported statistically significant improvements in SBP [33]. A study of an intensive RPM program focused on hypertension and deployed in an FQHC serving low-income Asian American patients reported that 96% of the patients improved their BP control following the intervention [34]. By contrast, this study found little change over time with respect to SBP or DBP, suggesting that patients may require an increased level of support while undergoing RPM to achieve meaningful reductions in BP.

Studies examining the feasibility and acceptability of RPM programs targeting hypertension in underserved patient populations have identified both positive and negative aspects of patients’ experiences. A study investigating knowledge of hypertension, engagement in care, and attitudes toward and experiences with SMBP in patients from 9 community health centers found that most patients (85%) reported having positive experiences with SMBP and that patients’ engagement with care increased significantly after SMBP [35]. The authors suggested that patients’ positive experiences were attributable to the fact that they were provided with education, training, and support while engaging in SMBP [35]. A mixed methods study conducted in Hmong and Latino adults with hypertension at an FQHC to examine patients’ perspectives about a mobile health–based care model including RPM found that patients found the program useful, especially if they were provided assistance with navigating technological challenges [36]. Sharing their BP data with the clinic felt empowering to some patients but entrapping to others [36]. A pilot study examining the feasibility and effectiveness of training high school students as health technology coaches to help medically underserved patients with hypertension found that, compared with the BPM-only group, the BPM-plus-health-coaching group had a higher frequency of self-monitoring, higher engagement and satisfaction, and better self-reported BP [37]. Patients expressed concerns regarding the inconsistency of results and reported that their health coaches helped them troubleshoot technical difficulties [37]. A systematic review of qualitative studies assessing patients’ experiences of RPM for chronic disease management found that RPM improved disease-specific knowledge, self-management, and decision-making and initiated earlier clinical assessment [38]. Patients’ concerns with RPM included a loss of interpersonal contact and increased personal responsibility [38]. A study of an intensive RPM program in an FQHC serving low-income Asian American patients identified low digital health literacy and a lack of in-language digital training as barriers to successful RPM [34].

The barriers to successful RPM uncovered in previous studies are similar to those identified in this study, in which patients expressed concerns about device accuracy and convenience and reported experiencing technical challenges with using their BPM. The body of evidence suggests that patient education and support are integral to successful RPM programs in low-resource settings. Therefore, the barriers identified in this study may be overcome by incorporating more intensive patient education and increasing patients’ access to personalized support from program staff. Future research should compare the different forms and intensities of patient education and support to discover which are most effective in underserved patient populations.

### Strengths and Limitations

This study has several key strengths. It is unique in that it examined an SMBP program that included training, education, and outreach and was implemented at an FQHC among underserved patients, many of whom were Latino and Spanish speaking. Our setting and population render our findings highly generalizable to other FQHCs; our findings can be leveraged to support further research, build capacity, and improve the digital health infrastructure of other FQHCs. The digital health program at FHCSD was designed under the premise that patient-provider relationships and health care professional support are essential for SMBP to improve hypertension outcomes. As such, the program was supported by digital health specialists who received direct training from the AHA on how to develop, implement, and manage a high-quality digital health program focused on SMBP. Our program included both one-on-one training on how to use a BPM and health education both in person at clinics and via telehealth encounters. Patients were also offered technical assistance with using their BPM.

This study also has important limitations. Although we assessed changes in SBP and DBP over time, our study design did not enable us to analyze how different factors (eg, demographic or clinical factors) influenced BP control status. Future studies with more robust designs should directly examine which patient characteristics are associated with BP control status. In addition, our data did not permit us to analyze which forms and intensities of patient education and support were most impactful for patients while engaging in RPM. Many patients rated the BPM favorably, but the low use rates suggest that the patients experienced challenges with using the BPM.

### Conclusions

This study demonstrated the acceptability of a simple, low-cost program for monitoring BP among patients at an FQHC. However, few patients were able to use the BPM at the rate that would result in device cost reimbursement. Such programs may not be financially feasible at scale if reimbursement does not occur. Given that RPM programs show promise in the FQHC setting, future research should focus on evaluating plans for sustaining costs among low-income, underserved patients so that this population is better supported in managing hypertension.

## Appendix

Multimedia Appendix 1 Hypertension and digital health literacy baseline survey template.

Multimedia Appendix 2 Template of the 3-month-postprogram survey.

Multimedia Appendix 3 Health education booklets provided to patients during training and onboarding. We collaborated with our local American Heart Association (AHA) branch to create these booklets for patients.

Multimedia Appendix 4 Digital health SMS text message campaign. These are the hypertension-related SMS text messages that were sent to the patients who opted into the SMS campaign.

## Abbreviations

AHA: American Heart Association
BP: blood pressure
BPM: blood pressure monitor
CPT: Current Procedural Terminology
DBP: diastolic blood pressure
EHR: electronic health record
FHCSD: Family Health Centers of San Diego
FQHC: federally qualified health center
RPM: remote patient monitoring
SBP: systolic blood pressure
SMBP: self-measured blood pressure
SUS: System Usability Scale
