# Supplementary material for: Digitally Based Blood Pressure Self-Monitoring Program That Promotes Hypertension Self-Management and Health Education Among Patients With Low-Income: Usability Study
Source: JMIR Hum Factors. 2023 Jul 24;10:e46313. doi: 10.2196/46313 (PMC10407769; doi:10.2196/46313)
Supplement: Multimedia Appendix 1 [file humanfactors_v10i1e46313_app1.docx]

**Hypertension and Digital Health Literacy Baseline Questions Template**

***This is a draft. The actual form will be filled out via Qualtrics.**

We will collect this information from you to describe our program population. We will also use this information to better understand your current digital health literacy. Please note that this information will be kept confidential. We will not disclose your personal information to a third party without your consent.

1. **Patient Information**
2. Today’s Date (Month/Day/Year): ______________
3. Type of encounter:
   1. In-clinic
   2. Virtual
4. What is your preferred language?

¿Cuál es su idioma preferido?

- 1. English
  2. Spanish
  3. Arabic
  4. Other: ______________

1. **High Blood Pressure**
2. Have you owned or do you currently own a blood pressure monitor at home?

¿Ha tenido o tiene actualmente un aparato de presión en casa?

- 1. Yes
  2. No

1. If yes, how often would you check your blood pressure at home?

¿Con qué frecuencia se toma la presión en casa?

- 1. Everyday
  2. About once a week
  3. About once a month
  4. Rarely
  5. Never

1. Are you currently taking blood pressure medication?

¿Está tomando actualmente medicamentos para la presión arterial?

- 1. Yes
  2. No

1. If yes, are you taking your medication as prescribed?

¿Está tomando su medicación según lo prescrito?

- 1. Yes
  2. Usually
  3. Sometimes
  4. No

1. Do you think that your blood pressure is controlled?

¿Cree usted que su presión está controlada?

- 1. Yes
  2. No
  3. I don’t know

1. How often can you sense that your blood pressure is too high just by the way you feel?

¿Con qué frecuencia puede sentir que su presión está demasiado alta solo por la forma en que se siente?

- 1. Always
  2. Sometimes
  3. Rarely
  4. Never

1. How would you rate your own health?

¿Cómo calificaría su propia salud?

- 1. Excellent
  2. Good
  3. Fair
  4. Poor

1. How often do you have thoughts of potentially being at risk for a heart attack or a stroke in the next few years?

¿Con qué frecuencia tiene pensamientos de estar potencialmente en riesgo de sufrir un ataque cardíaco o un derrame cerebral en los próximos años?

- 1. Always
  2. Sometimes
  3. Never

1. Do you think adding salt to foods can raise blood pressure?

¿Cree que agregar sal a los alimentos puede aumentar la presión?

- 1. Yes
  2. No
  3. I don’t know

1. Do you think a diet that consists of mainly fresh fruits and vegetables helps to lower blood pressure?

¿Cree que una dieta que se compone principalmente de frutas y verduras frescas ayuda a bajar la presión?

- 1. Yes
  2. No
  3. I don’t know

1. Do you think walking or exercising for at least 30 minutes a day helps to lower blood pressure?

¿Cree que caminar o hacer ejercicio al menos 30 minutos al día ayuda a reducir la presión?

- 1. Yes
  2. No
  3. I don’t know

1. Do you think smoking and drinking can raise blood pressure?

¿Cree que fumar y beber pueden elevar la presión?

- 1. Yes
  2. No
  3. I don’t know

1. **Digital Health Technology Literacy**
2. Do you own a smartphone?

¿Tiene un teléfono inteligente?

1. Yes
2. No
3. If yes, do you know how to download/install phone applications?

¿Sabe cómo descargar / instalar aplicaciones telefónicas?

1. Yes
2. No
3. Do you have a computer, laptop, or iPad at home?

¿Tiene una computadora, laptop o iPad en casa?

1. Yes
2. No
3. Do you have internet and/or Wi-Fi at home?

¿Tienes internet y / o wifi en casa?

1. Yes
2. No
3. Do you know where online health information is available on the Internet? For example, the Centers for Disease Control and Prevention (CDC), the U.S. Food and Drug Administration, etc.

¿Sabe dónde está disponible la información de salud en línea en Internet? Por ejemplo, los Centros para el Control y la Prevención de Enfermedades (CDC), la Administración de Drogas y Alimentos de EE. UU., Etc.

- 1. Yes
  2. No

Please rate the following on a scale of “Not at all familiar” “Somewhat familiar” and “Completely familiar”:

Califique lo siguiente en una escala de "Nada familiar", "Algo familiar" y "Completamente familiar":

1. How familiar are you with the following items?
   1. Online Patient Portals

Portales de pacientes en línea

- 1. Mobile Heath Applications

Aplicaciones de salud móviles

- 1. Wearable Devices

Aparatos portátiles

- 1. Telehealth

1. How familiar are you with the following items?
   1. Keyboard

Teclado

- 1. App Store

Tienda de aplicaciones

- 1. Username/Password

Nombre de usuario y contraseña

- 1. Operating System (e.g., Microsoft Windows)

Sistema operativo (por ejemplo, Microsoft Windows)

Please rate the following on a scale of “Not at all confident” “Somewhat confident” and “Completely confident”:

Califique lo siguiente en una escala de "Nada seguro", "Algo seguro" y "Completamente seguro":

1. How confident do you feel...?
   1. Using a computer in general
   2. Using touchscreen
   3. Finding information online
   4. Creating an online account
