# Supplementary material for: Digitally Based Blood Pressure Self-Monitoring Program That Promotes Hypertension Self-Management and Health Education Among Patients With Low-Income: Usability Study
Source: JMIR Hum Factors. 2023 Jul 24;10:e46313. doi: 10.2196/46313 (PMC10407769; doi:10.2196/46313)
Supplement: Multimedia Appendix 2 [file humanfactors_v10i1e46313_app2.docx]

**3 Month Post Program Survey Questions Template**

We will collect this information from you to gather feedback on how our patients liked the monitor. Please note that this information will be kept confidential. We will not disclose your personal information to a third party without your consent.

1. **Patient Information**
2. Today’s Date (Month/Date/Year): ______________
3. Which encounter did patient receive?
   1. In-clinic
   2. Virtual
4. Did patient receive the SMS campaign?
   1. Yes
   2. No
5. **Withings BPM Connect**

Please rate the following on a scale of “Strongly disagree” “Disagree” “Neutral” “Agree” and “Strongly agree”:

1. I think that I would like to use this system frequently. (ORIGINAL)

I think that I would like to use the Withings BPM frequently. (MODIFIED)

Creo que me gustaría usar Withings BPM con frecuencia. (TRANSLATION)

1. I found the system unnecessarily complex. (ORIGINAL)

I found the Withings BPM complicated to use. (MODIFIED)

Encontré el Withings BPM complicado de usar. (TRANSLATION)

1. I thought the system was easy to use. (ORIGINAL)

I found the Withings BPM easy to use. (MODIFIED)

Encontré el Withings BPM fácil de usar. (TRANSLATION)

1. I think that I would need the support of a technical person to be able to use this system. (ORIGINAL)

I think that I would need the support of a digital health specialist to be able to use the Withings BPM. (MODIFIED)

Creo que necesitaría el apoyo de un especialista en salud digital para poder utilizar el Withings BPM. (TRANSLATION)

1. I found the various functions in this system were well integrated. (ORIGINAL)

I found the Withings BPM's measurements easy to read. (MODIFIED)

Encontré las medidas de Withings BPM fáciles de leer. (TRANSLATION)

1. I would imagine that most people would learn to use this system very quickly. (ORIGINAL)

I would imagine that most people would learn to use the Withings BPM very quickly. (MODIFIED)

Me imagino que la mayoría de la gente aprendería a usar Withings BPM muy rápidamente. (TRANSLATION)

1. I felt very confident using the system. (ORIGINAL)

I felt very confident using the Withings BPM. (MODIFIED)

Me sentí muy seguro usando Withings BPM. (TRANSLATION)

1. I needed to learn a lot of things before I could get going with this system. (ORIGINAL)

I needed to learn a lot of things before I could use the Withings BPM. (MODIFIED)

Necesitaba aprender muchas cosas antes de poder usar el Withings BPM. (TRANSLATION)

Please rate the following on a scale of “Very bad” “Bad” “Neutral” “Good” and “Very good”:

1. How would you rate the blood pressure monitor?

¿Cómo calificaría el aparato?

1. Would you recommend the blood pressure monitor to other patients?

¿Recomendaría este aparato de la presión a otros pacientes?

- 1. Yes
  2. No

1. Do you think the blood pressure monitor is helping you better manage your hypertension? Why or why not?

¿Cree que el aparto le está ayudando a controlar mejor su presión? ¿Por qué o por qué no?

________________________________________

1. Are you willing to continue using the blood pressure monitor for another 3 months? We will continue to monitor your results and notify your provider if there are any issues.

¿Está dispuesto a continuar usando el aparato de la presión por otros 3 meses? Continuaremos monitoreando sus resultados y notificaremos a su doctor si hay algún problema.

________________________________________

References:

[System Usability Scale (SUS) | Usability.gov](https://www.usability.gov/how-to-and-tools/methods/system-usability-scale.html)

1. I think that I would like to use this system frequently.
2. I found the system unnecessarily complex.
3. I thought the system was easy to use.
4. I think that I would need the support of a technical person to be able to use this system.
5. I found the various functions in this system were well integrated.
6. I thought there was too much inconsistency in this system. (removed)
7. I would imagine that most people would learn to use this system very quickly.
8. I found the system very cumbersome to use. (removed)
9. I felt very confident using the system.
10. I needed to learn a lot of things before I could get going with this system.
