# Supplementary material for: Digitally Based Blood Pressure Self-Monitoring Program That Promotes Hypertension Self-Management and Health Education Among Patients With Low-Income: Usability Study
Source: JMIR Hum Factors. 2023 Jul 24;10:e46313. doi: 10.2196/46313 (PMC10407769; doi:10.2196/46313)
Supplement: Multimedia Appendix 3 [file humanfactors_v10i1e46313_app3.pdf]

FAMILY HEALTH CENTERS
OF SAN DIEGO

Digital Health Program

How to measure your blood pressure at home

TARGET:BP™ | American Heart Association. | AMA

Follow these steps for an accurate blood pressure measurement

1. PREPARE

Avoid ca eine, smoking and exercise for 30 minutes before measuring your blood pressure.

Wait at least 30 minutes after a meal.

If you're on blood pressure medication, measure your BP *before* you take your medication.

Empty your bladder beforehand.

Find a quiet space where you can sit comfortably without distraction.

2. POSITION

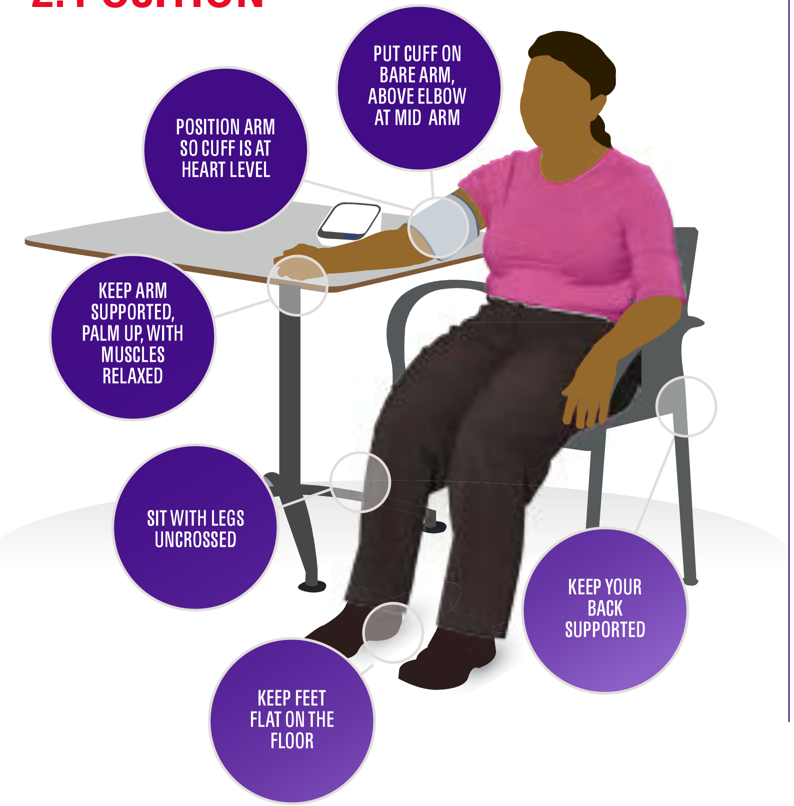

3. MEASURE

Rest for ve minutes while in position before starting.

Take two or three measurements, one minute apart, twice daily for seven days.

Keep your body relaxed and in position during measurements.

Sit quietly with no distractions during measurements—avoid conversations, TV, phones and other devices.

Record your measurements when nished.

Content provided by

AMA | MAPBP™

©2020 American Medical Association. All Rights Reserved.
10/20 MRG15940-6B

This Prepare, position, measure handout was adapted with permission of the American Medical Association and The Johns Hopkins University. The original copyrighted content can be found at https://www.ama-assn.org/ama-johns-hopkins-blood-pressure-resources.

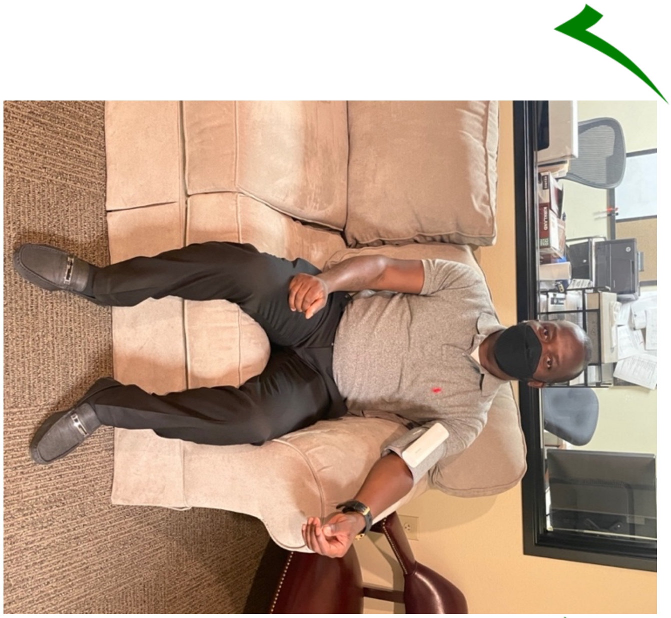

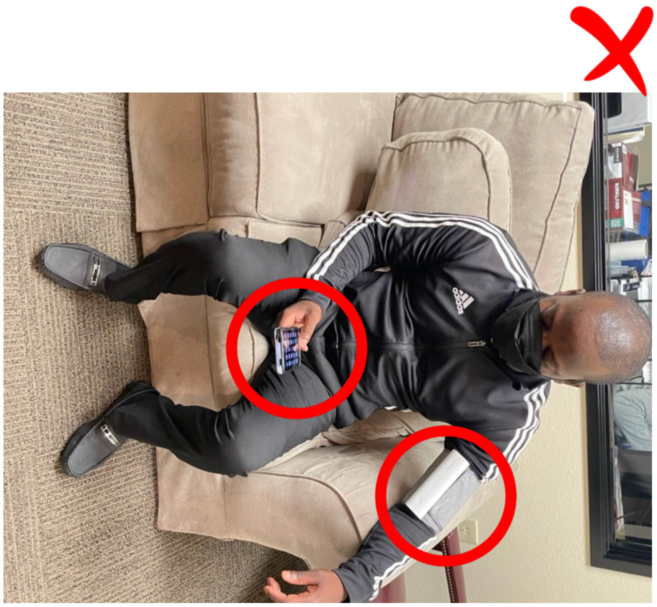

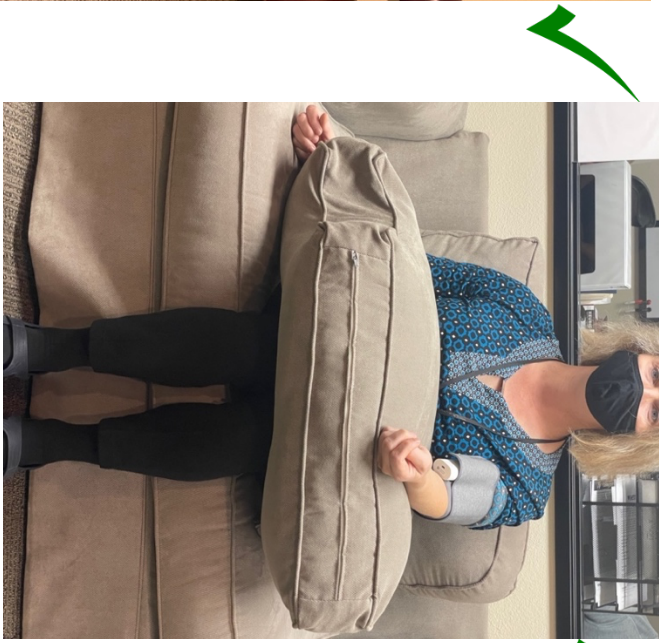

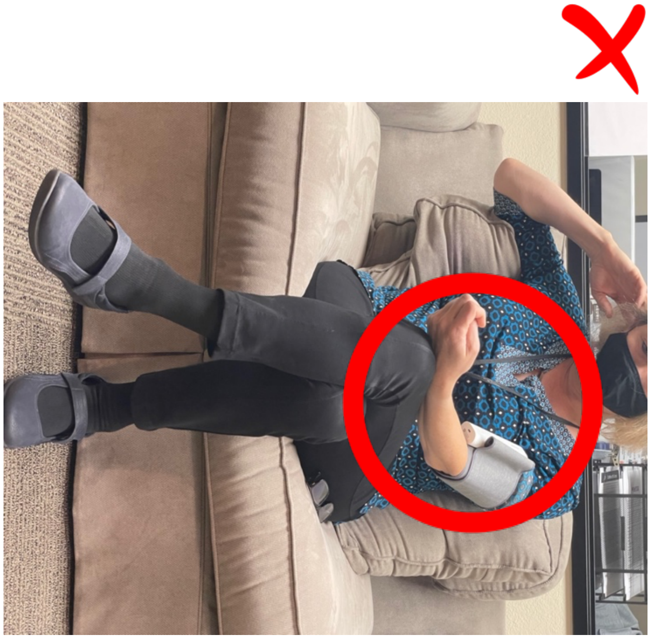

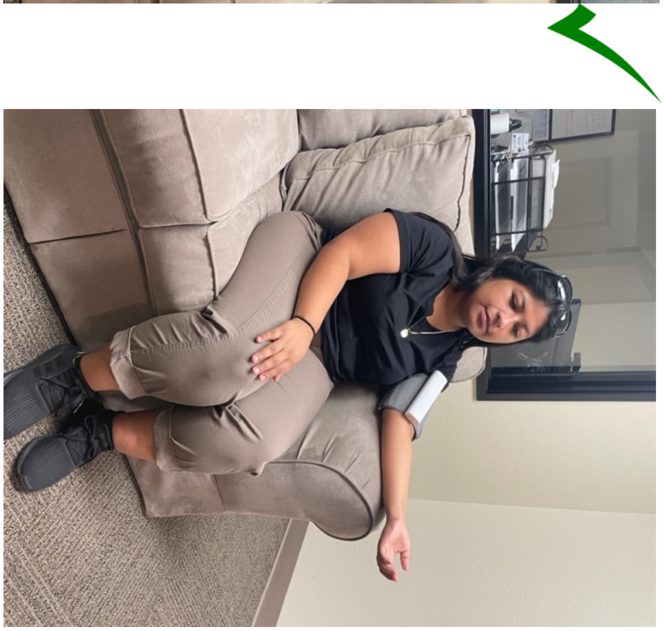

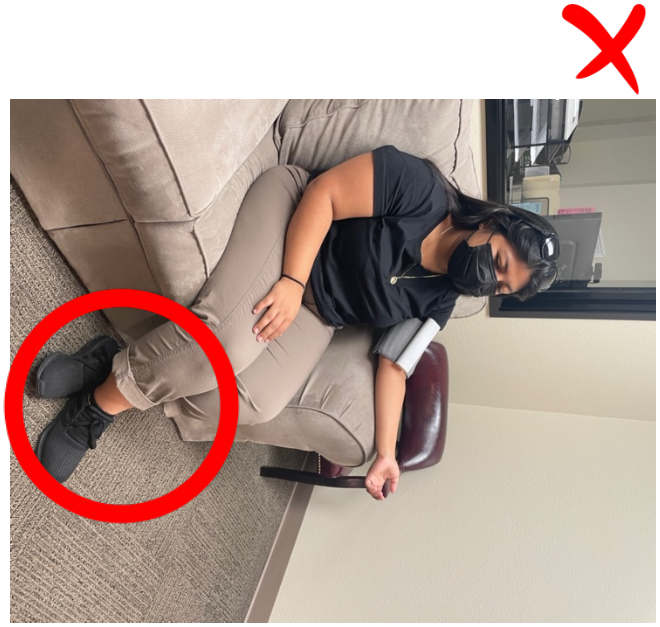

What is High Blood Pressure?

Blood pressure is the force of blood pushing against blood vessel walls. It is measured in millimeters of mercury (mm Hg).

High blood pressure (HBP) means the pressure in your arteries is higher than it should be. Another name for high blood pressure is hypertension.

Blood pressure is written as two numbers, such as 112/78 mm Hg. The top (systolic) number is the pressure when the heart beats. The bottom (diastolic) number is the pressure when the heart rests between beats.

Normal blood pressure is below 120/80 mm Hg. If you're an adult and your systolic pressure is 120 to 129, and your diastolic pressure is less than 80, you have **elevated blood pressure**. **High blood pressure** is a systolic pressure of 130 or higher,or a diastolic pressure of 80 or higher, that stays high over time.

High blood pressure usually has no signs or symptoms. That's why it is so dangerous. But it can be managed.

Nearly half of the American population over age 20, has HBP, and many don't even know it. Not treating high blood pressure is dangerous. High blood pressure increases the risk of heart attack and stroke.

Make sure you get your blood pressure checked regularly and treat it the way your health care provider advises.

BLOOD PRESSURE CATEGORY	SYSTOLIC mm Hg (upper number)		DIASTOLIC mm Hg (lower number)
NORMAL	LESS THAN 120	and	LESS THAN 80
ELEVATED	120-129	and	LESS THAN 80
HIGH BLOOD PRESSURE (HYPERTENSION) STAGE 1	130-139	or	80-89
HIGH BLOOD PRESSURE (HYPERTENSION) STAGE 2	140 OR HIGHER	or	90 OR HIGHER
HYPERTENSIVE CRISIS (consult your doctor immediately)	HIGHER THAN 180	and/ or	HIGHER THAN 120

Am I at higher risk of developing HBP?

There are risk factors that increase your chances of developing HBP. Some you can control, and some you can't.

Those that can be controlled are:

- Cigarette smoking and exposure to secondhand smoke
- Diabetes
- Being obese or overweight
- High cholesterol
- Unhealthy diet (high in sodium, low in potassium, and drinking too much alcohol)
- Physical inactivity

Factors that can't be modified or are difficult to control are:

- Family history of high blood pressure
- Race/ethnicity
- Increasing age
- Gender (males)
- Chronic kidney disease
- Obstructive sleep apnea

Socioeconomic status and psychosocial stress are also risk factors for HBP. These can affect access to basic living needs, medication, health care providers, and the ability to adopt lifestyle changes.

American Heart Association.

What Can I Do To Improve My Blood Pressure?

Modification	Recommendation	Approximate SBP Reduction Range
Weight reduction	Maintain normal body weight (BMI=18.5-24.9 kg/m^2)	5 mm Hg
DASH eating plan	Diet rich in fruits, vegetables, low fat dairy and reduced in fat	11 mm Hg
Restrict sodium intake	<1500 mg of sodium per day	5-6 mm Hg
Physical activity	Be more physically active. Aim for at least 90 to 150 minutes of aerobic exercise per week.	5-8 mm Hg
Moderation of alcohol consumption	No more than 2 drinks/day for men and 1 drink/day for women	4 mm Hg

BP = Blood pressure, BMI = Body mass index, SBP = Systolic blood pressure, DASH = Dietary Approaches to Stop Hypertension

Ranges According to 2017 Hypertension Clinical Practice Guidelines

Recommendations for Treatment and Management of Hypertension

American Heart Association.

Shelf-Stable Healthy Eating

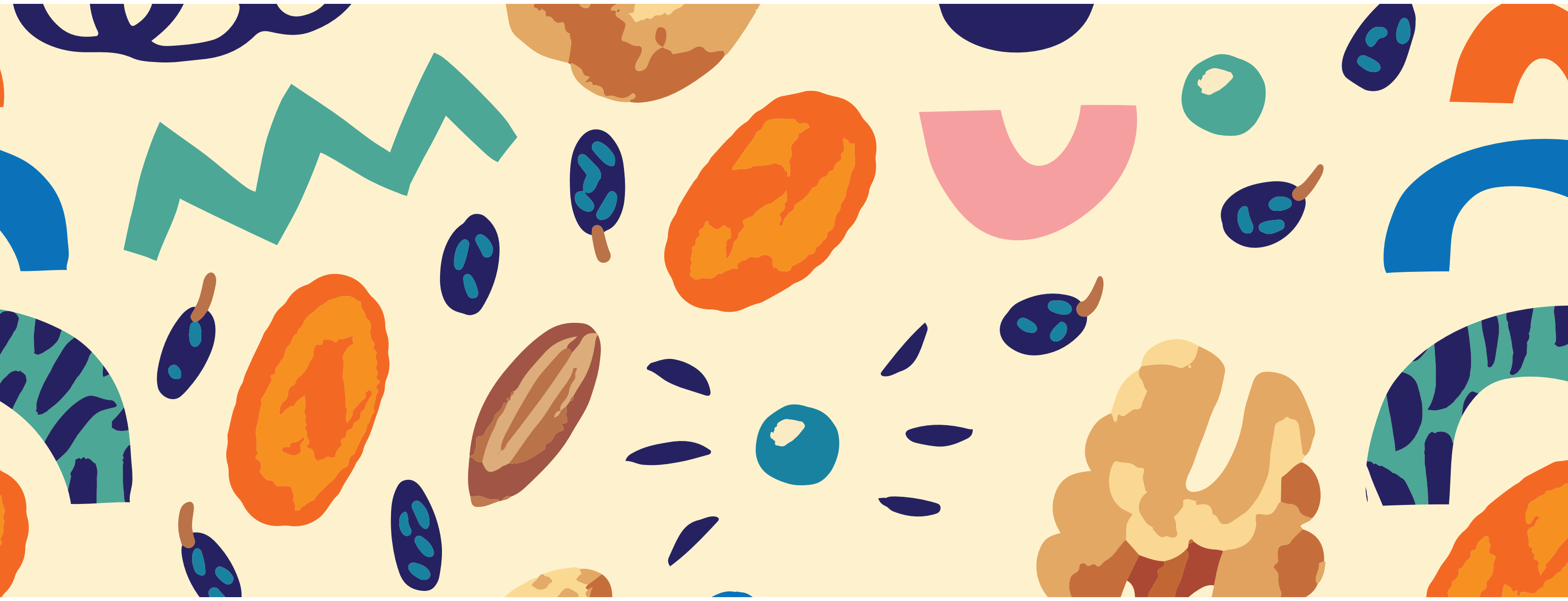

IF YOU'RE CONCERNED ABOUT HAVING HEALTHY FOODS ON HAND WHILE LIMITING YOUR EXPOSURE TO CROWDS, THESE HEART-HEALTHY RECIPES CAN ALL BE MADE WITH SHELF-STABLE INGREDIENTS SUCH AS:

- Canned, frozen and dried fruits and vegetables (low or no salt and sugar options).
- Canned meats like light tuna or white meat chicken (salt free), packed in water.
- Frozen chicken breast is safe for up to 1-year in a freezer set to zero degrees or below (store as air-tightly as possible to preserve maximum freshness).
- Dried beans and legumes (or canned with no salt added).
- Dried whole grains like brown rice and quinoa.
- Dried herbs and spices.
- Shelled eggs are safe 3-5 weeks and unopened egg substitute is safe up to 1 year in the refrigerator.

American Heart Association®
Healthy for Good™

CHOOSE YOUR OWN WORKOUT

Circuits can be a great way to work out without any special equipment

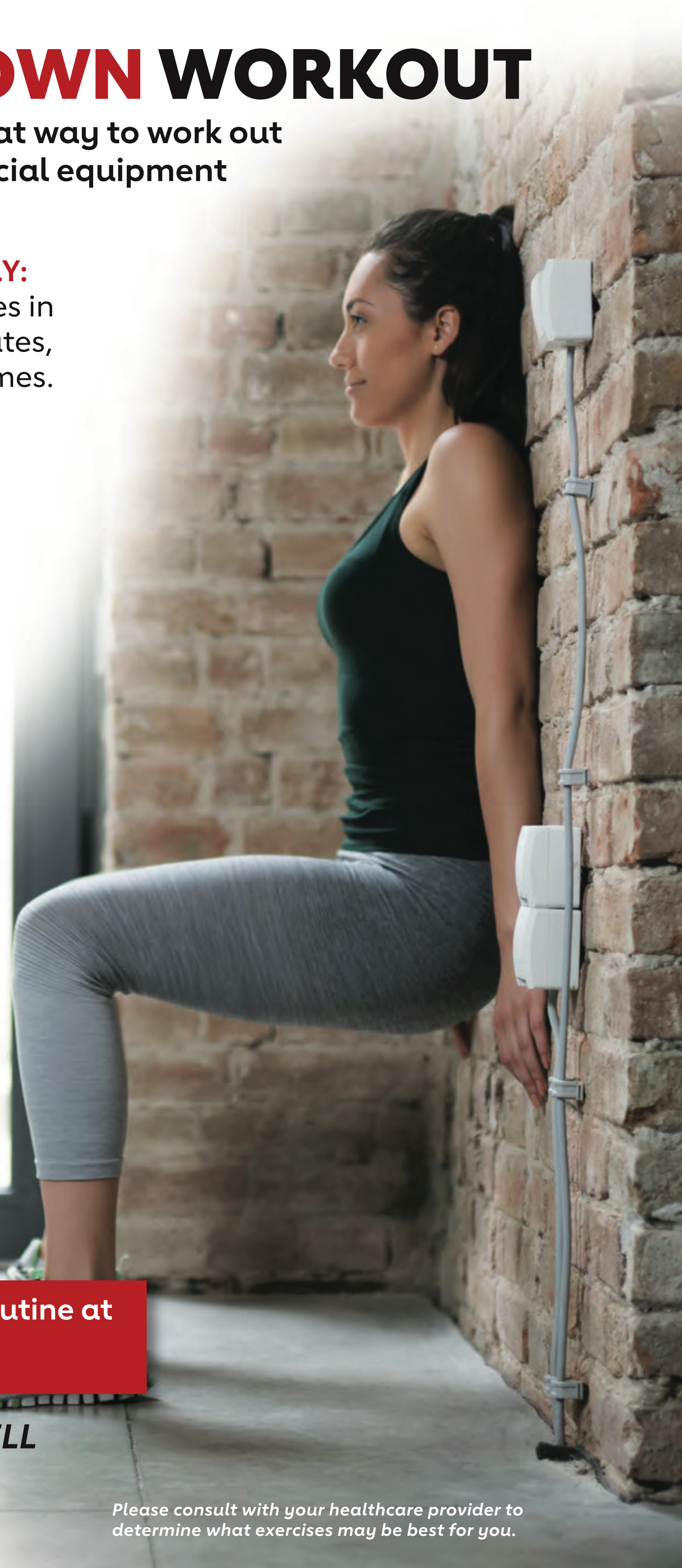

TO BUILD YOUR CIRCUIT, CHOOSE 3-4 EXERCISES FROM EACH CATEGORY:
Alternate cardio and strength exercises in short bursts of 30 seconds and 3 minutes, then repeat the circuit two to three times.

CARDIO EXERCISES

- Jumping Jacks
- Squat Jumps
- Jogging or Marching in Place
- Stair-Climbing or Step-Ups
- High Knees
- Mountain Climbers
- Star Jumps
- Burpees

STRENGTHENING AND STABILITY EXERCISES

- Plank and Side Plank
- Pushups
- Sit-Ups or Crunches
- Hip Lift or Bridge Postition
- Tricep Dips on a Chair
- Lunges
- Squats or Chair Position
- Wall Sits

Learn more ways to add activity to your routine at
heart.org/HealthyForGood

EAT SMART ***MOVE MORE*** ***BE WELL***

© Copyright 2020 American Heart Association, Inc., a 501(c)(3) not-for-profit. All rights reserved.
Healthy for Good is a trademark. Unauthorized use prohibited. 3/20DS15967

Please consult with your healthcare provider to determine what exercises may be best for you.

American Heart Association®
Healthy for Good™

FIGHT STRESS WITH HEALTHY HABITS

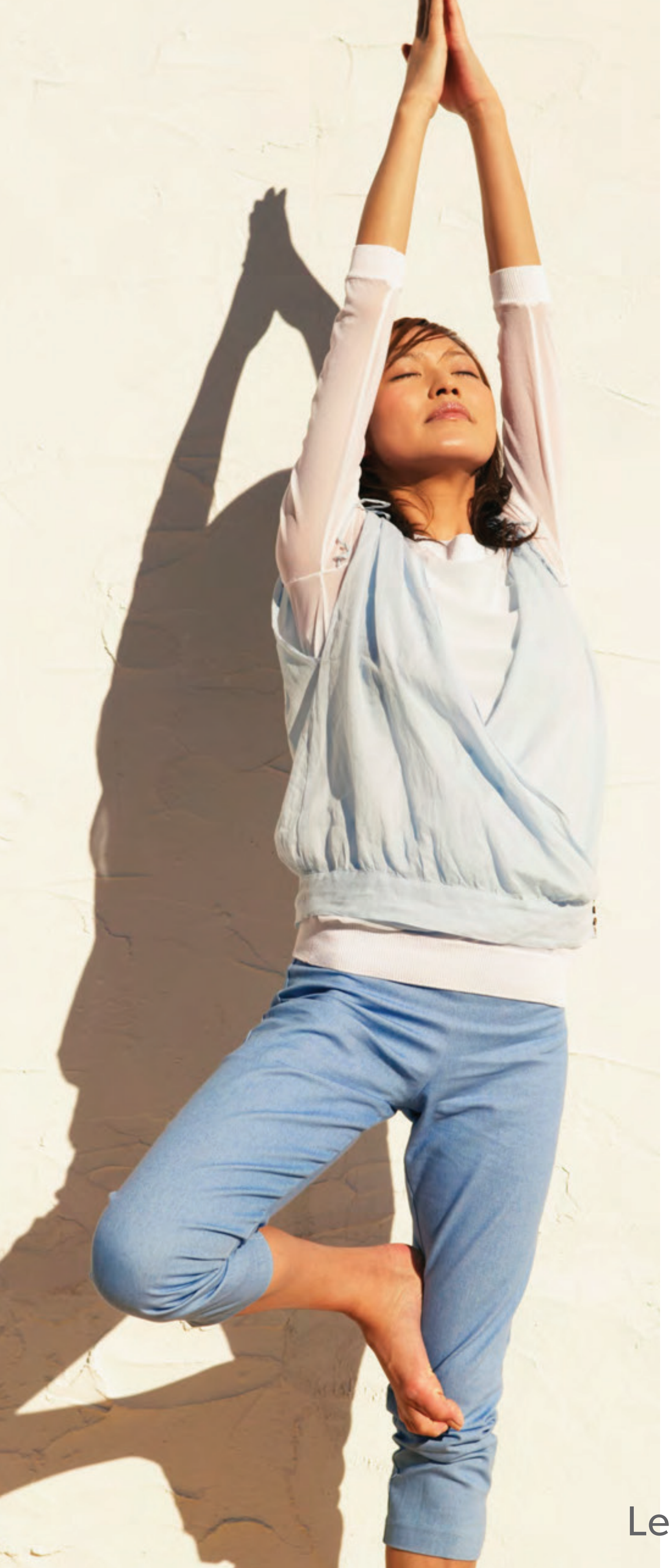

1. **Slow down.**
Plan ahead and allow enough time to get the most important things done without having to rush.

2. **Snooze more.**
Try to get seven to nine hours of sleep each night. To fight insomnia, add mindfulness and activity.

3. **Let worry go.**
The world won't end if a few things fall off of your plate. Give yourself a break and just breathe.

4. **Laugh it up.**
Laughter makes us feel good. Don't be afraid to laugh out loud, even when you're alone.

5. **Get connected.**
A daily dose of friendship is great medicine. Make time to call friends or family so you can catch up.

6. **Get organized.**
Use "to do" lists to help you focus on your most important tasks and take big projects one step at a time.

7. **Practice giving back.**
Volunteer your time or spend time helping out a friend. Helping others helps you.

8. **Be active every day.**
Exercise can relieve mental and physical tension. Find something you think is fun and stick with it.

9. **Give up the bad habits.**
Too much alcohol, tobacco or caffeine can increase blood pressure. Cut back or quit to decrease anxiety.

10. **Lean into things you can change.**
Make time to learn a new skill, work toward a goal, or to love and help others.

Learn more at **heart.org/HealthyForGood**

EAT SMART MOVE MORE BE WELL

© Copyright 2020 American Heart Association, Inc. DS15662 2/20

American Heart Association.

What do I need to know about COVID-19 if I have high blood pressure?

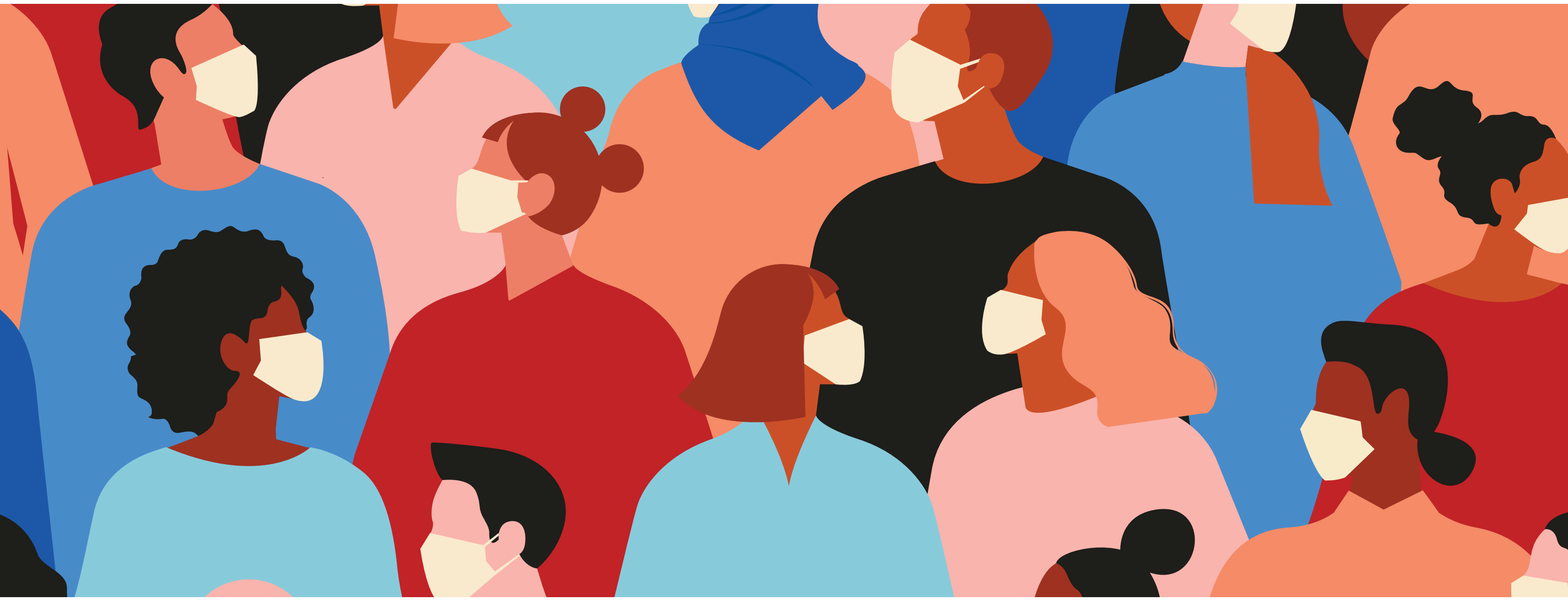

BECAUSE OF YOUR CONDITION, YOU FACE HIGHER RISKS OF COMPLICATIONS FROM COVID-19 IF YOU CONTRACT THE DISEASE, WHICH MAKES CAUTION EXTREMELY IMPORTANT.

Do everything possible to avoid exposure to the coronavirus. Staying away from other people is critically important — especially if you are in your 60s or older. And don't forget the safety basics:

- **handwashing with soap and water for at least 20 seconds**
- **not touching surfaces**
- **cleaning surfaces diligently**

The CDC has a full list of recommended precautions. In addition, continue taking all your medications as prescribed, including ACE inhibitors and ARBs. These medications do not increase your risk of contracting COVID-19.

If you are diagnosed with COVID-19, you should be fully evaluated before adding or removing any treatments. And high blood pressure patients and diabetes patients should both keep in mind that your overall medical condition is much better if your blood pressure and diabetes are optimally controlled.

Coronavirus precautions for patients & others facing higher risks

American Heart Association.

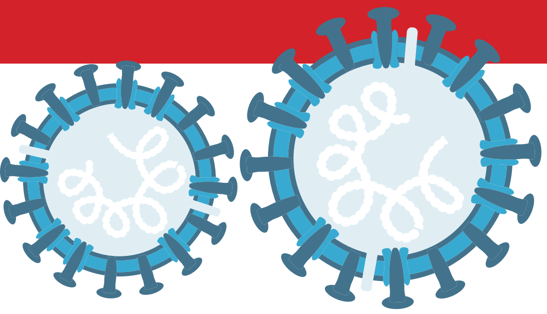

The American Heart Association is advising caution and preparation for elderly people with coronary heart disease or hypertension because they are more likely to be infected and to develop more severe symptoms. Stroke survivors may face increased risk for complications if they get COVID-19.

Get Prepared at Home

Make sure you can reach your doctor quickly.

If you live alone, **gather a list of support contacts who you might call on if needed.**

Take stock of your medications. Make sure you have enough for an extended time. Also figure out how you would get refills if you couldn't leave home.

Make sure you have plenty of food, beverage and hygiene supplies for yourself, your family and your pets.

What if you have symptoms of coronavirus?

Common symptoms include **fever** and **cough**. Contact your health care provider if you have these symptoms. **If you experience shortness of breath or other heart attack or stroke warning signs, call 911.**

- Make sure appropriate caregivers are available at home.
- Ensure there's a separate bedroom where the patient can recover without sharing immediate space with others.
- Set up some basic rules for making sure the person being isolated can get food and other necessities with minimal risk.

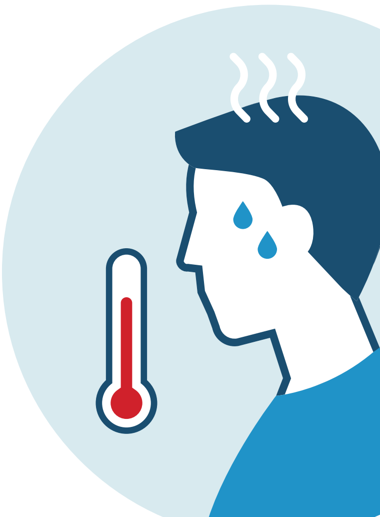

Locally supported by

American Heart Association®
