# Supplementary material for: Digitally Based Blood Pressure Self-Monitoring Program That Promotes Hypertension Self-Management and Health Education Among Patients With Low-Income: Usability Study
Source: JMIR Hum Factors. 2023 Jul 24;10:e46313. doi: 10.2196/46313 (PMC10407769; doi:10.2196/46313)
Supplement: Multimedia Appendix 4 [file humanfactors_v10i1e46313_app4.docx]

| **WEEK 1** | **Day 1** | **Day 2** | **Day 3** | **Day 4** | **Day 5** | **Day 6** | **Day 7** |
| --- | --- | --- | --- | --- | --- | --- | --- |
| **Time:**  9:00 AM | **SMS (Content Message 1):**  *Regularly monitoring your blood pressure will help your medical provider know how you are doing which can help to improve your care.*  *Monitorear regularmente su presión ayudará a su doctor a saber cómo está, lo que puede ayudar a mejorar su salud.* | SMS (Content Message 2):  *Tip: Avoid caffeine, smoking and exercise for 30 min before measuring blood pressure.*  *Tip: Evite la cafeína, fumar y hacer ejercicio 30 minutos antes de medir su presión.* | **SMS (Content Message 3):**  *Next time you check your blood pressure, share your results with family and friends! This will help keep you on track.*  *La próxima vez que se tome la presión, ¡comparta sus resultados con familiares y amigos! Esto lo(a) mantendrá al tanto de su presión.* | SMS (Content Message 4):  *Did you know? Distractions and talking can add a higher inaccurate blood pressure reading? Remember to stay still and silent when you measure.*  *¿Sabía que las distracciones y las conversaciones pueden resultar en una medida incorrecta de la presión? Recuerde permanecer quieto (a) y en silencio cuando mida.* | **SMS (Content Message 5):** *If your blood pressure is too high, plan to reduce your salt intake, reduce stress, and exercise more.*  *Si su presión es demasiado alta, planee reducir su consumo de sal, reducir el estrés y hacer más ejercicio*. | SMS (Content Message 6):  *Taking blood pressure medication? It is important to measure your blood pressure before you take your blood pressure medication.*  *¿Toma medicamentos para la presión? Es importante medir su presión antes de tomar su medicamento para la presión.* | **SMS (Content Message 7):**  *Tip: Wait at least 30 minutes after a meal to measure your blood pressure.*  *Tip: Espere al menos 30 minutos después de una comida para medir su presión.* |
| **WEEK 2** | **Day 8** | Day 9 | **Day 10** | Day 11 | **Day 12** | Day 13 | **Day 14** |
| **Time:**  9:00am | **SMS (Content Message 8):**  *Sometimes you can feel OK but still have blood pressure that is higher than your target range. Measure regularly to know if you are on track!*  *A veces puede sentirse bien pero aun así tener la presión alta. ¡Mida regularmente para saber si está al tanto de su presión!* | SMS (Content Message 9):  *To get accurate blood pressure readings, measure your blood pressure on an empty bladder.*  *Para obtener medidas precisas de la presión, mida su presión con la vejiga vacía.* | **SMS (Content Message 10):**  *Monitor your blood pressure regularly. This can help you and your medical provider take action to protect your heart, eyes, and kidney health.*  *Tome su presión con regularidad. Esto puede ayudar a usted y a su doctor a tomar decisiones adecuadas para proteger la salud de su corazón, ojos y riñones*. | SMS (Content Message 11):  *It is not always easy to stay on your blood pressure target. If you are off target, plan for things you can change in the coming week.*  *No siempre es fácil mantener la presión en la categoría recomendada. Si está fuera de su objetivo, planifique las cosas que puede cambiar la próxima semana.* | **SMS (Content Message 12):**  *Checking your blood pressure regularly is a big step towards feeling better and spending more time doing the things you want to do!*  *Tomar su presión con regularidad es un gran paso para sentirse mejor y pasar más tiempo haciendo las cosas que desea hacer.* | SMS (Content Message 13):  *When taking your blood pressure, remember to sit up straight with your legs uncrossed.*  *Cuando se tome la presión, recuerde sentarse con la espalda recta y sin cruzar las piernas.* | **SMS (Content Message 14):**  *Checking blood pressure regularly is a big step towards preventing complications in the future.*  *Tomar la presión con regularidad es un gran paso para prevenir complicaciones en el futuro.* |
| **WEEK 3** | **Day 15** | Day 16 | **Day 17** | Day 18 | **Day 19** | Day 20 | **Day 21** |
| **Time:**  9:00am | **SMS (Content Message 15):**  *Protect your heart by keeping your blood pressure under control. Healthy heart, happy you!*  *Proteja su corazón manteniendo su presión bajo control. Corazón sano, vida sana.* | SMS (Content Message 16):  *Need to sleep better? Try spending some time relaxing each day. This can help manage stress and promote healthy blood pressure.*  *¿Necesita dormir mejor? Intente pasar algún tiempo relajándose todos los días. Esto puede ayudar a controlar el estrés y promover una presión saludable.* | **SMS (Content Message 17):**  *Lack of sleep can negatively affect your mood, stress, and blood pressure. Try to get at least 7-8 hours of sleep each night.*  *La falta de sueño puede afectar negativamente su estado de ánimo, estrés y presión. Trate de dormir al menos 7-8 horas cada noche.* | SMS (Content Message 18):  *Getting 7-8 hours of sleep each night gives you enough energy to work out and stick to healthy eating. This can also help lower your blood pressure.*  *Dormir 7 a 8 horas cada noche le da suficiente energía para hacer ejercicio y mantener una alimentación saludable. Esto también puede ayudar a reducir su presión.* | **SMS (Content Message 19):**  *Sleep is SO much more than feeling rested. Not getting enough is associated with heart diseases and getting sick. Make sure to prioritize it every night!*  *Dormir es mucho más que descansar un rato. No dormir lo suficiente puede provocar enfermedades y problemas cardíacos ¡Asegúrate de priorizarlo todas las noches!* | SMS (Content Message 20):  *Going outside in natural sunlight for at least 30 minutes each day will help to regulate your sleep patterns, which can help lower blood pressure. Try eating your lunch outside or by walking outdoors.*  *Salir al aire libre al menos 30 minutos todos los días ayudará a regular su sueño, lo que puede ayudar a reducir la presión. Intente comer su almuerzo al aire libre o salir a caminar al aire libre.* | **SMS (Content Message 21):**  *To promote healthy blood pressure, try creating a healthy sleep routine. Try to go to bed at the same time each night.*  *Para mantener la presión saludable, intente crear una rutina de sueño saludable. Trate de acostarse a la misma hora cada noche.* |
| **WEEK 4** | **Day 22** | Day 23 | **Day 24** | Day 25 | **Day 26** | Day 27 | **Day 28** |
| **Times:**  9:00am | **SMS (Content Message 22):**  *Avoid caffeine and alcohol before bedtime. This can help promote better sleep and healthy blood pressure.*  *Evite la cafeína y el alcohol antes de acostarse. Esto puede ayudar a promover un mejor sueño y una presión saludable.* | SMS (Content Message 23):  *Fun fact: You can get a better night’s rest by engaging in exercise. Getting enough quality sleep can help lower blood pressure.*  *Dato curioso: Puede descansar mejor por la noche haciendo ejercicio. Dormir lo suficiente puede ayudar a reducir la presión.* | **SMS (Content Message 24):**  *Try gradually increasing your exercise. For example, start at 20 minutes a day a few times a week, and work up to 30 minutes a day. This can help control your blood pressure.*  *Intente aumentar su ejercicio. Por ejemplo, comience con 20 minutos al día algunas veces a la semana y aumente hasta 30 minutos al día. Esto puede ayudar a controlar su presión.* | SMS (Content Message 25):  *Challenge: Watching your favorite show? Try walking in place or stretching. This can help lower your blood pressure.*  *Desafío: ¿Está viendo su programa favorito? Intente caminar o estirar mientras ve su programa. Esto puede ayudar a reducir su presión.* | **SMS (Content Message 26):**  *Challenge: Try connecting with someone, such as a family member or friend to keep you motivated in exercising. This can promote healthy blood pressure.*  *Desafío: Intente conectarse con alguien, como un familiar o un amigo, para mantenerse motivado en el ejercicio. Esto puede promover una presión saludable.* | SMS (Content Message 27):  *Get moving! Doing a brisk (fast-paced) walk 30 minutes every day can help lower your blood pressure.*  *¡Mueve tu cuerpo! Hacer una caminata enérgica (de ritmo rápido) 30 minutos todos los días puede ayudar a reducir la presión.* | **SMS (Content Message 28):**  *Try including muscle-strengthening exercises a couple of times a week. Using weights such as dumbbells and even a 2 Liter water bottle can help lower your blood pressure.*  *Intente ejercicios de fortalecimiento muscular un par de veces a la semana. El uso de pesas e incluso una botella de agua de 2 litros puede ayudar a reducir la presión.* |
| **WEEK 5** | **Day 29** | Day 30 | **Day 31** | Day 32 | **Day 33** | Day 34 | **Day 35** |
| **Time:**  9:00am | **SMS (Content Message 29):**  *Mix up your workout routine! For example, 15 min of dancing and 15 min of walking. Physical activity is important for having healthy blood pressure.*  *¡Mezcle su rutina de ejercicio! Por ejemplo, 15 minutos de baile y 15 minutos de caminata. La actividad física es importante para tener una presión saludable.* | SMS (Content Message 30):  *Find something you enjoy. For example, if you like the beach, get a jog in while enjoying the outdoor scenery. This can help promote healthy blood pressure.*  *Haga algo que disfrute. Por ejemplo, si le gusta la playa, trote mientras disfruta del paisaje al aire libre. Esto puede ayudar a promover una presión arterial saludable.* | **SMS (Content Message 31):**  *Activities such as swimming, stair-climbing, and bicycling are great for keeping your blood pressure at a healthy level.*  *Las actividades como nadar, subir escaleras y andar en bicicleta son excelentes para mantener su presión en un nivel saludable.* | SMS (Content Message 32):  *What is your favorite song or album? Try dancing to it for some exercise. This can promote healthy blood pressure.*  *¿Cuál es su canción o álbum favorito? Intente bailar para hacer algo de ejercicio. Esto puede promover una presión saludable.* | **SMS (Content Message 33):**  *Enjoy milk? Try consuming low-fat dairy products to reduce your sodium intake to maintain healthy blood pressure.*  *¿Le gusta la leche? Intente consumir productos lácteos bajos en grasa para reducir su consumo de sal y mantener una presión saludable.* | SMS (Content Message 34):  *Feeling hungry? Sometimes thirst feels like hunger. Choose water over sugary drinks such as soda to manage your blood pressure.*  *¿Tiene hambre? A veces, la sed se siente como hambre. Elija agua en lugar de bebidas azucaradas para controlar su presión.* | **SMS (Content Message 35):**  *Lower your sodium intake by eating less red meat and more fish and skinless poultry. This can help lower your blood pressure.*  *Reduzca su consumo de sal comiendo menos carnes rojas y más pescado y pollo sin piel. Esto puede ayudar a reducir su presión.* |
| **WEEK 6** | **Day 36** | Day 37 | **Day 38** | Day 39 | **Day 40** | Day 41 | **Day 42** |
| **Time:**  9:00am | **SMS (Content Message 36):**  *Try limiting your alcohol to no more than 1-2 drinks a day. This can help lower your blood pressure.*  *Intente limitar su consumo de alcohol a no más de 1 a 2 bebidas al día. Esto puede ayudar a reducir su presión.* | SMS (Content Message 37:  *Instead of adding salt, try adding fresh lemon juice or fresh black pepper to your vegetables and fish. This simple seasoning swap can help your blood pressure.*  *En lugar de agregar sal, intente agregar jugo de limón fresco o pimienta negra fresca a sus verduras y pescado. Este simple cambio de condimento puede ayudar a bajar la presión.* | **SMS (Content Message 38):**  *Aim for whole grains such as whole-grain tortillas and bread. Whole grains contain more fiber compared to refined grains, which are better for your heart health.*  *Trate de consumir cereales integrales, igual tortillas y pan integrales. Los cereales integrales contienen más fibra en comparación con los cereales refinados, que son mejores para la salud de su corazón.* | SMS (Content Message 39):  *Processed packaged foods contain a lot of salt. Try snacking on fresh, whole foods such as fruits and vegetables to maintain healthy blood pressure.*  *Los alimentos envasados ​​contienen mucha sal. Intente comer bocadillos frescos e integrales, como frutas y verduras, para mantener una presión saludable.* | **SMS (Content Message 40):**  *Nutrition labels tell you how much sodium (salt) contains. Stay under 1,500mg of sodium a day to maintain healthy blood pressure.*  *Las etiquetas nutricionales le indican la cantidad de sal que contiene. Manténgase por debajo de los 1500 mg de sal al día para mantener una presión saludable.* | SMS (Content Message 41):  *Sodium can sneak into many foods you eat, such as cheese and olives. Check the sodium content on the food labels to control your blood pressure.*  *La sal puede estar presente en muchos alimentos que consume, como el queso y las aceitunas. Verifique el contenido de sal en las etiquetas de los alimentos para controlar su presión.* | **SMS (Content Message 42):**  *To manage your blood pressure, listen to your favorite song, whether it relaxes you or pumps you up. Doesn't it feel good?!*  *Para controlar su presión, escuche su canción favorita, ya sea que lo relaje o lo estimule. ¿No se siente bien?* |
| **WEEK 7** | **Day 43** | Day 44 | **Day 45** | Day 46 | **Day 47** | Day 48 |  |
| **Time:**  9:00am | **SMS (Content Message 43):**  *It takes a team! Get the support you need to stay active - family and friends can help you meet your goals of maintaining healthy blood pressure.*  *¡Se necesita un equipo! Obtenga el apoyo que necesita para mantenerse activo: la familia y los amigos pueden ayudarlo a alcanzar sus objetivos de mantener una presión saludable.* | SMS (Content Message 44):  *Calm your mind by stretching for 5 minutes! This can help maintain healthy blood pressure. Ideas for positions: touch your toes, reach hands to the sky, stretch to the right and left.*  *¡Calme su mente estirando durante 5 minutos! Esto puede ayudar a mantener una presión saludable. Ideas: toque los dedos de los pies, extienda las manos hacia el cielo, estire hacia la derecha y hacia la izquierda.* | **SMS (Content Message 45):**  *Be on the lookout! Unhealthy behaviors like eating too much or not exercising can be early signs of stress and affect your blood pressure negatively.*  *Los comportamientos poco saludables como comer demasiado o no hacer ejercicio pueden ser signos tempranos de estrés y afectar negativamente la presión.* | SMS (Content Message 46):  *Fight stress by talking with friends and family. A great way to do this is by taking a walk outside together, which is great for your blood pressure and overall well-being.*  *Combata el estrés hablando con amigos y familiares. Una excelente manera de hacerlo es dar un paseo juntos al aire libre, lo cual es excelente para la presión y el bienestar general.* | **SMS (Content Message 47):**  *Trying to reduce your stress? Try not to pack too many tasks into every moment. Allowing for moments to be in the present can help your blood pressure.*  *¿Tratando de reducir su estrés? Trate de no hacer demasiados quehaceres al mismo tiempo. Haga las cosas una a la vez y esto puede ayudar a reducir su presión.* | SMS (Content Message 48):  *Relaxing is key in controlling your blood pressure! Take 15 minutes each day to sit and breathe deeply.*  *¡Relajarse es clave para controlar su presión! Tómese 15 minutos cada día para sentarse y respirar profundamente.* |  |
